# Maternal Exercise Rescues Embryonic Osteogenesis Impaired due to POLG Mutation Through a Potential Apelin‐ATF4 Axis

**DOI:** 10.1002/advs.77029

**Published:** 2026-08-07

**Authors:** Song Ah Chae, Ryan C. Riddle, Zhihua Jiang, Chaeyoung Shin, Min Du, Jun Seok Son

**Affiliations:** ^1^ Laboratory of Perinatal Exercise Genomics Department of Obstetrics Gynecology & Reproductive Sciences University of Maryland School of Medicine Baltimore Maryland USA; ^2^ Department of Anesthesiology University of Maryland School of Medicine Baltimore Maryland USA; ^3^ Division of Musculoskeletal Sciences Department of Orthopaedics University of Maryland School of Medicine Baltimore Maryland USA; ^4^ Research and Development Service Baltimore VA Medical Center Baltimore Maryland USA; ^5^ Nutrigenomics & Growth Biology Laboratory Department of Animal Sciences Washington State University Pullman Washington USA; ^6^ Department of Pharmacology & Physiology University of Maryland School of Medicine Baltimore Maryland USA

**Keywords:** bone, exercise, fetus, pregnancy, skeletal muscle

## Abstract

For proper locomotion, bone and muscle development must be tightly coordinated. Skeletal muscle secretes myokines that affect tissues, including bone. We previously showed that maternal exercise (ME) enhances fetal muscle development through apelin signaling. However, whether mitochondrial dysfunction impairs fetal skeletal development and whether ME mitigates these defects remain unclear. Heterozygous POLG mutant (*Polg* ^mut/+^) mice were used to examine the effects of ME and apelin signaling on fetal osteogenesis. Pregnant *Polg* ^mut/+^ females underwent treadmill exercise, and apelin receptor knockdown (*Apj* ^KD^) mice and maternal apelin supplementation were used to evaluate apelin signaling. POLG mutation impaired fetal osteogenesis and disrupted skeletal morphology. RNA‐seq revealed downregulation of pathways related to cytoskeletal organization and mitochondrial function. ME was associated with upregulation of osteogenesis and development. ME increased apelin abundance in maternal‐fetal circulation and tissues, including fetal muscle and bone. Importantly, apelin administration increased fetal osteogenesis in POLG mutation, whereas *Apj* ^KD^ reduced osteogenic signaling in fetal bone and myogenic gene expression in fetal muscle. Apelin also enhanced mitochondrial respiration in fetal bone‐derived osteogenic cells. Mechanistically, ME‐induced apelin signaling was associated with increased mitochondrial biogenesis and enhanced ATF4‐RUNX2 regulatory signaling. Collectively, these findings suggest that ME‐induced apelin signaling contributes to fetal skeletal development under mitochondrial dysfunction.

## Graphical abstract

Maternal exercise (ME) increases apelin abundance across maternal and fetal tissues and is associated with improved fetal osteogenesis under POLG mutation‐induced mitochondrial dysfunction. Apelin‐APJ signaling is linked to enhanced mitochondrial function, Akt phosphorylation, and ATF4‐RUNX2 association, supporting coordinated fetal bone remodeling through a potential apelin‐ATF4 axis.

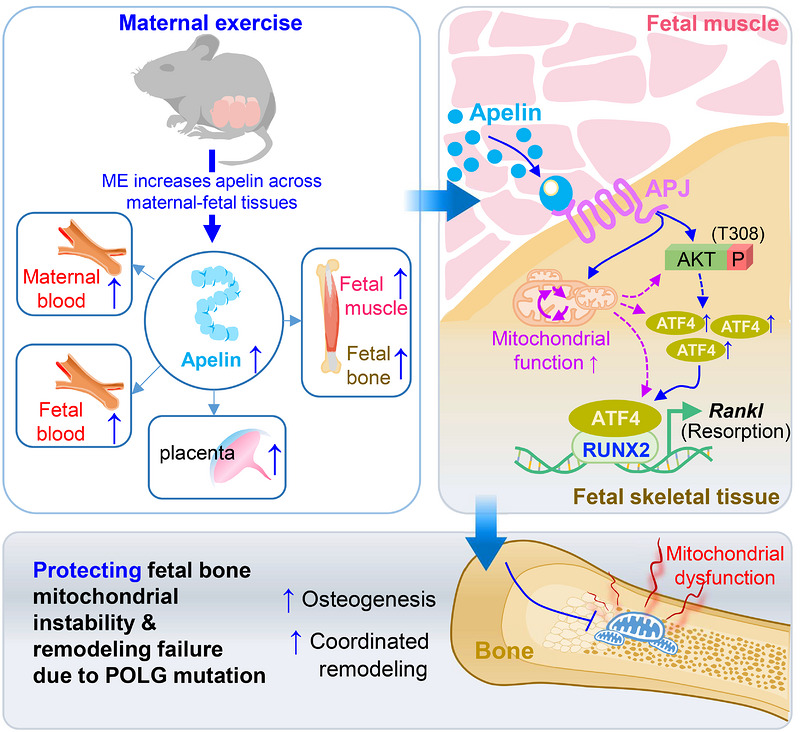

## 1 Introduction

Skeletal muscle comprises about 40% of the human body mass and plays a crucial role in maintaining mechanical and physiological homeostasis, particularly in preserving the balance between bone and muscle remodeling [1, 2]. In addition, proper skeletal muscle development significantly reduces the risk of fall‐related fractures [3]. Epidemiological and meta‐analysis data support a strong association between low muscle mass and reduced bone formation in both children and adults [4, 5, 6, 7, 8]. We previously reported that muscle development in the fetus and offspring can be stimulated by exercise interventions during pregnancy [9, 10]. Despite these findings, the mechanisms underlying the protective effect of maternal exercise (ME) on fetal bone formation through maternal‐fetal compartments‐derived secretory molecules remain poorly understood.

Mutations in mitochondrial DNA (mtDNA) polymerase γ (POLG) gene result in the expression of mutant proteins that lack polymerase proofreading activity in mitochondria, which impairs mitochondrial DNA maintenance and compromises oxidative metabolic functions [11]. Through regulating mitochondrial DNA maintenance and biogenesis, POLG is essential for proper mammalian embryogenesis [12]. Indeed, mitochondria have been reported to directly regulate osteogenesis by facilitating mineral deposition in bone collagen [13]. Emerging evidence suggests that mitochondrial metabolism during development is tightly regulated by epigenetic mechanisms controlling genes involved in mitochondrial biogenesis [14, 15, 16]. Our recent studies showed that maternal exercise (ME) promotes the secretion of exercise‐related hormones such as apelin, which in turn enhances skeletal muscle mitochondrial biogenesis and activity in the fetus [9, 10]. Considering the intricate coordination between fetal muscle and bone development, we hypothesized that ME improves embryonic bone formation via stimulating mitochondrial biogenesis through apelin signaling and mitochondrial biogenesis by apelin signaling.

Apelin, an endogenous peptide ligand derived from the *Apln* gene, is widely recognized as an exercise‐induced myokine and adipokine, responsible for energy metabolism and cardiovascular functions [17, 18]. Its receptor, APJ, encoded by *Aplnr*, is a G protein‐coupled receptor expressed in multiple metabolically active tissues, including skeletal muscle and bone [17, 18]. Upon apelin binding, APJ activates intracellular signaling cascades such as AMPK, PI3K‐AKT, and ERK1/2 pathways, which regulates mitochondrial function, cellular survival, and anabolic signaling [17, 19]. Within skeletal muscle, apelin enhances oxidative metabolism and mitochondrial biogenesis, supporting contractile performance and metabolic flexibility [20]. In bone, APJ is expressed in osteoblast lineage cells and osteoclast precursors, where its activation promotes osteoblast proliferation and survival while modulating RANKL‐mediated osteoclast differentiation [21, 22]. Emerging evidence suggests that the apelin‐APJ axis serves as a molecular bridge between muscle and bone, which integrates exercise‐induced metabolic modulation during skeletal remodeling processes [23, 24]. Thus, apelin‐mediated signaling represents a critical endocrine and paracrine mechanism that coordinates mitochondrial metabolism in muscle with anabolic and anti‐resorptive pathways in bone, supporting overall musculoskeletal homeostasis.

Activation of transcription factor 4 (ATF4), also known as cAMP‐response element binding protein 2 (CREB2), is critical for the survival, proliferation, and differentiation of osteoblasts in adult bone tissues [25]. During early‐life skeletal development, ATF4 contributes to osteoblast maturation by regulating genes involved in extracellular matrix production and collagen synthesis, processes essential for proper bone matrix formation [26, 27]. In addition to ATF4, runt‐related transcription factor 2 (RUNX2) is another key transcription factor for osteoblast differentiation and is considered a master regulator of bone development [28, 29]. Importantly, ATF4 cooperates functionally with RUNX2, which together form a transcriptional regulatory complex that enhances the expression of osteoblast‐specific genes, including osteocalcin (OCN), coordinating osteoblast maturation and bone matrix deposition during bone formation [30, 31].

In this study, we used POLG mutant mice as a model of impaired mitochondrial metabolism to test whether mitochondrial dysfunction impairs fetal osteogenesis and whether ME‐induced apelin signaling mitigates these defects. We hypothesized that ME‐induced apelin signaling improves fetal bone development and formation through enhanced co‐regulation between ATF4 and RUNX2 following APJ activation.

## 2 Methods

### 2.1 Animals

All animal protocols were conducted in AAALAC‐accredited facilities following the ARRIVE guidelines and approved by the Institutional Animal Care and Use Committee (IACUC) of the University of Maryland School of Medicine (#AUP‐00000151) and Washington State University (#ASAF‐6704). Heterozygous DNA polymerase γ gene (gene: *Polg*, protein: PolG) mutated mice carrying the *PolgA* ^D257A^ allele (*PolgA* ^D257A^; 10 to 12 weeks old), purchased from Jackson laboratory (#017341; Bar Harbor, ME, USA), were bred with heterozygous POLG males to generate homozygous POLG mutant (*Polg* ^mut^) and wild‐type (WT) fetuses as littermates. Fetal samples were collected on embryonic (e) day 18.5. Maternal mice were housed under a 12 h light/dark cycle with ad libitum access to chow control diet and water.

In a separated animal study, ten to twelve weeks old heterozygous POLG mutant females were randomly assigned to two groups: (1) maternal control (M‐Ctrl) and (2) maternal exercise (M‐Ex). All female mice were acclimated to treadmill exercise for one week prior to mating. After mating with age‐matched heterozygous POLG mutant male mice, pregnant mice in the M‐Ex group performed daily treadmill exercise training every morning throughout pregnancy, according to our previously established method [32]. Homozygous POLG mutant fetuses were viable at e18.5, allowing fetal tissue collection and biochemical analyses at this developmental stage.

We further utilized another separated animal study using ten to twelve weeks old heterozygous POLG mutant female mice, which were randomly assigned to two groups: (1) maternal PBS injection (M‐PBS) and (2) maternal apelin administration (M‐APN). Apelin was intraperitoneally (i.p.) administered daily with [Pyr1]apelin‐13 (AAPPTec, Louisville, KY, USA) at 0.5 µmol/kg daily from e1.5 to e16.5, as described in our previous studies [9, 10, 33]. Simultaneously, PBS i.p. injection was performed for serving as a control group. Then, fetal samples (homozygous POLG mutant fetuses were viable) were collected at e18.5 for biochemical analyses.

To generate global apelin receptor (APJ) knockdown (KD) fetuses, we used our *Apj* floxed (*Apj* ^fl/fl^) mice [9, 10] crossed with *β‐actin* ^Cre^ mice (#019099, Jackson Laboratory). *Apj* ^fl/+^:*β‐actin* ^Cre(±)^ female mice were bred with WT male mice to generate 1) *Apj* ^fl/+^:*β‐actin* ^Cre(±)^ and 2) WT fetuses as littermates. For characterization of the *Apj* ^KD^ model, *Apj* mRNA expression and APJ protein levels were analyzed in e18.5 fetal bone and fetal muscle by qPCR and immunoblotting, respectively. Fetal samples were collected at e18.5 for biochemical analyses.

### 2.2 Fetal Tissue Collection

Fetal tissues were collected at e18.5. For skeletal tissue analysis, fetal forelimbs and hindlimbs were carefully dissected under a stereomicroscope. Skin, distal paw tissue, and surrounding muscle were completely removed, and only the remaining skeletal elements were collected for biochemical analyses. For fetal muscle analysis, limb muscle tissues were isolated separately from the same fetuses after removal of skin and connective tissues, ensuring no contamination from skeletal elements. All samples were immediately snap frozen in liquid nitrogen and stored at −80°C until further analysis. Given the developmental stage at e18.5, the isolated skeletal tissues may contain both mineralizing bone and cartilaginous components.

### 2.3 Endurance Treadmill Exercise Training

Daily treadmill exercise training was performed according to our previous treadmill exercise protocol for pregnant mice [32]. Prior to mating, all female mice were subjected to flat degree treadmill running for adaptation three times per week for one week. After mating, pregnant mice in the exercise group completed daily treadmill exercise training every morning for one hour: 1) 10 min warming up (intensity: 5 m/min), 2) 40 min main exercise (10–14 m/min), and 10 min cooling down (5 m/min).

### 2.4 Seahorse Extracellular Flux Analysis

Fetal bone‐derived osteogenic cells were isolated from e18.5 fetuses of wild‐type C57BL/6J pregnant mice. Briefly, after removal of surrounding soft tissues, fetal skeletal tissues were sequentially digested with 0.05% trypsin‐EDTA followed by 1 mg/mL collagenase P, and isolated cells were seeded onto Seahorse XF miniplates. Then, the fetal bone‐derived osteogenic cells were treated with either 100 nm [Pyr1]apelin‐13 or vehicle for 24 h as previously described [10]. Oxygen consumption rate (OCR) was measured using the Seahorse XF HS Mini Analyzer (Agilent Technologies, Santa Clara, CA, USA), under basal conditions and after sequential injection of oligomycin, FCCP, and rotenone/antimycin A. OCRs were normalized to total cell count and mitochondrial respiration parameters, including basal respiration, ATP‐linked respiration, maximal respiration, spare respiratory capacity, proton leak, and non‐mitochondrial respiration, were calculated according to the manufacturer's instructions.

### 2.5 Histology

For histological analysis of fetal muscle and bone, hindlimbs were fixed in 4% paraformaldehyde (PFA) and embedded in paraffin. Five µm‐thickness fetal limbs were prepared for hematoxylin and eosin (H&E) staining, as described previously [9, 10, 33]. For fetal muscle morphometric analysis, myofiber cross‐sectional areas (CSAs) were measured from H&E‐stained fetal skeletal muscle sections using ImageJ by tracing individual myofiber boundaries, and mean CSA values were calculated for each biological replicate. For immunofluorescence (IF) analyses, sections were incubated with primary antibodies against OCN (#16157‐1‐AP; RRID: AB_2878225) or TRAP (#11594‐1‐AP; RRID: AB_2877781) from Proteintech (Rosemont, IL, USA), or apelin (#PA5‐114860; RRID: AB_2899496) from Invitrogen (Rockford, IL, USA), followed by appropriate fluorescence‐conjugated secondary antibody (Alexa Fluor 488 anti‐rabbit IgG antibody; #406416; RRID:AB_2563203; BioLegend; San Diego, CA, USA), and nuclei were counterstained with DAPI (#H‐1200‐10; Vector Laboratory, Inc.; Newark, CA, USA). Fluorescence intensity was analyzed across the indicated regions in each section by ImageJ software.

### 2.6 RNA‐Seq

Alternative polyadenylation with whole transcriptome termini site sequencing (WTTS‐seq) was performed as previously described [9, 10, 33, 34]. We utilized four limb musculoskeletal without skin to extract RNAs. One µg total RNA was used for enriching poly‐A RNA, followed by size selection and asymmetric amplification of cDNA, as previously detailed [9, 10, 33, 34]. After trimming, alignment was performed and mapped to the reference genome (Mus_musculus.GRCm38.fa). Differentially expressed genes (DEGs) and gene ontology (GO) analyses were conducted. DEGs were defined as those with |log2 fold change| ≥ 1 and adjusted *p* value (FDR) < 0.01. Functional enrichment analyses were performed using gene ontology (GO) and gene set enrichment analysis (GSEA). In addition, weighted gene co‐expression network analysis (WGCNA) was conducted to identify gene modules associated with genotype and to determine hub genes within biologically relevant modules.

### 2.7 qPCR

Total RNA was extracted from fetal muscle or bone using TRIzol reagent (Invitrogen, Grand Island, NY, USA). cDNA was synthesized using RNA and the iScript cDNA Synthesis Kit (Bio‐Rad, Hercules, CA, USA). Gene expression was qualified using PowerUp SYBR Green Master Mix (Applied Biosystems, Waltham, MA) on a real‐time quantitative PCR machine (QuantStudio 3, Applied Biosystems). Relative gene expression levels were calculated using the 2^−ΔΔCt^ method, normalized to *18S* rRNA and *Actb*. Primer sequences used in this study are listed in Table S1.

### 2.8 Immunoblotting

Proteins were extracted using lysis buffer (100 mm Tris‐HCl pH 6.8, 2.0% SDS, 20% glycerol, 0.02% bromophenol blue, 5% 2‐mercaptoethanol, 100 mm NaF, and 1 mm Na_3_VO_4_), and concentrations of protein lysates were measured using the Pierce BCA Protein Assay Kit (Thermo Fisher Scientific, Waltham, MA, USA). Primary antibodies used were: OCN (#16157‐1‐AP; RRID: AB_2878225), ALPL (#11187‐1‐AP; RRID: AB_2305523), RUNX2 (#20700‐1‐AP; RRID: AB_2722783), RANKL (#23408‐1‐AP; RRID: AB_2879273), TRAP (#11594‐1‐AP; RRID: AB_2877781), p‐Akt (Thr308; #29163‐1‐AP; RRID: AB_2918241), Akt (#10176‐2‐AP; RRID: AB_2224574), p‐mTOR (Ser2448; #67778‐1‐Ig; RRID: AB_2889842), mTOR (#66888‐1‐Ig; RRID: AB_2882219), p‐P70S6K (Thr389; #28735‐1‐AP; RRID: AB_2918197), P70S6K (#14485‐1‐AP; RRID: AB_2269787), PGC‐1α (#66369‐1‐Ig; RRID: AB_2828002), TFAM (#22586‐1‐AP; RRID: AB_11182588), APJ (#20341‐1‐AP; RRID: AB_2878676), ATF4 (#10835‐1‐AP; RRID: AB_2058600), GAPDH (#10494‐1‐AP; RRID: AB_2263076), and β‐tubulin (#66240‐1‐Ig; RRID: AB_2881629) were purchased from Proteintech (Rosemont, IL, USA). VDAC (#4661; RRID: AB_10557420) was purchased from Cell Signaling Technology (Danvers, MA, USA). Apelin (#PA5‐114860; RRID: AB_2899496) was obtained from Invitrogen (Rockford, IL, USA). For secondary antibodies, IRDye 800CW goat anti‐rabbit and IRDye 680RD goat anti‐mouse antibodies were purchased from LI‐COR Biosciences (Lincoln, NE, USA). The ChemiDoc MP Imaging System (Bio‐Rad, Hercules, CA, USA) was used to detect the target proteins.

### 2.9 Co‐Immunoprecipitation

Total proteins were extracted in lysis buffer (0.75% NP‐40, 1 mm DTT) containing protease inhibitor (#4693116001, Roche cOmplete, Roche Diagnostics Deutschland GmbH, Mannheim, Germany). Lysates were precleaned with protein A magnetic particles (#MJA‐102, EcoMag, Bioclone Inc., San Diego, CA, USA) at 4°C for 1 h [35]. Two µg anti‐ATF4 primary antibody (#10835‐1‐AP; RRID: AB_2058600; Proteintech, Rosemont, IL, USA) was used to incubate with 500 µg of total protein overnight at 4°C with gentle rotation. Precleaned protein A magnetic beads were then added and rotated for 2 h at room temperature. Beads were washed three times with PBS and bound proteins were eluted for immunoblotting. Co‐IP signals were interpreted relative to input protein levels, and differences in total protein abundance were considered when evaluating changes in ATF4‐RUNX2 association.

### 2.10 Methylated DNA Immunoprecipitation PCR

Genomic DNA was extracted from e18.5 fetal bone using lysis buffer containing 10 mm Tris‐HCl, 1.0 mm EDTA, 20 mm NaCl, 1% SDS, and 20 mg/mL proteinase K, based on our previous protocols [9, 33]. Isolated DNA was diluted with TE buffer, sonicated, and denatured. Two µg of denatured DNA was incubated with either IgG or anti‐5mC antibody in IP buffer containing 10 mm sodium phosphate, 140 mm NaCl, and 0.05% Triton X‐100. Immunoprecipitation was then performed using Protein A Magnetic Beads (EcoMagTM; #MJA‐102, Bioclone Inc., San Diego, CA, USA). The immunoprecipitated DNA was quantified using PowerUp SYBR Green Master Mix (Applied Biosystems, Waltham, MA, USA) on a real‐time quantitative PCR system (QuantStudio 3, Applied Biosystems). IgG was used for normalization as previously described [9, 33]. Primer sequences are listed in Table S1.

### 2.11 Statistics

All experimental data presented as mean ± s.e.m. Sample size represents the number of independent biological replicates, as indicated in the figure legends. Statistical analyses were performed using GraphPad Prism 9 (GraphPad Software, San Diego, CA, USA) and SPSS Software (Version 21; IBM Corp., Armonk, NY, USA). For comparisons between two groups, unpaired two‐tailed Student's *t*‐test was used. For multiple group comparisons, one‐way analysis of variance (ANOVA) followed by appropriate post–hoc tests was applied. For RNA‐seq analysis, differential gene expression was determined using predefined thresholds (|log2 fold change| and false discovery rate, FDR), as described above. *P* values < 0.05 were considered statistically significant.

## 3 Results

### 3.1 RNA‐Seq Analysis Reveals that POLG Mutation Disrupts Fetal Musculoskeletal Development

**FIGURE 1:**
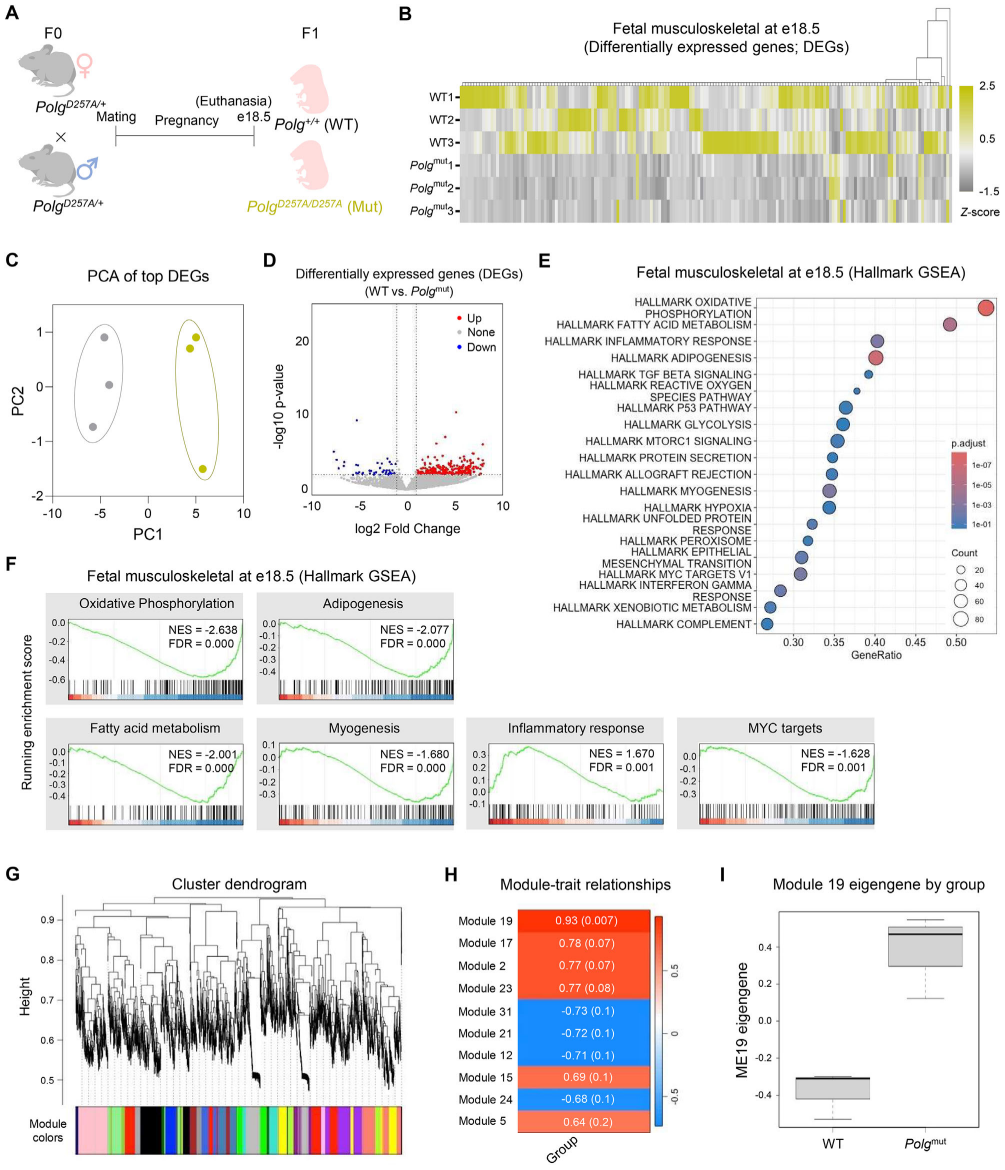
Whole‐Transcriptome Termini Site Sequencing (WTTS‐seq) analysis of e18.5 fetal hindlimb with POLG mutation. (A) Experimental design for investigating fetal POLG mutation. (B) Heatmap of differentially expressed genes (DEGs) in fetal musculoskeletal tissues at e18.5 comparing WT and POLG mutation. Values are shown as *Z*‐scores. (C) Principal component analysis of top DEGs showing separation between WT and POLG mutant fetal musculoskeletal tissues. (D) Volcano plot of DEGs between WT and POLG mutation. Upregulated genes are shown in red and downregulated genes in blue. (E) Hallmark gene set enrichment analysis (GSEA) of fetal musculoskeletal tissues at e18.5. Dot size represents gene count, and color indicates adjusted *p* value. (F) Representative enrichment plots for selected Hallmark pathways identified by GSEA, including oxidative phosphorylation, adipogenesis, fatty acid metabolism, myogenesis, inflammatory response, and MYC targets. Normalized enrichment score (NES) and false discovery rate (FDR) are indicated in each plot. (G) WGCNA cluster dendrogram showing gene modules identified from fetal musculoskeletal transcriptomic data. (H) Module‐trait relationship heatmap showing correlations between module eigengenes and genotype group. Correlation coefficients and corresponding *p* values are shown in each cell. (I) Box plot of the Module 19 module eigengene by group, showing differential module expression between WT and POLG mutant fetal musculoskeletal tissues.

RNA‐seq analysis was performed to determine how impaired mitochondrial metabolism, modeled using POLG mutant mice, alters transcriptional programs governing fetal musculoskeletal development at e18.5, by using whole transcriptome termini site (WTTS) sequencing previously described [9, 10, 33]. To determine whether mitochondrial dysfunction disrupts fetal bone formation and resorption, we used a POLG mutation mouse model (Figure 1A). Genotyping analysis confirmed the presence of wild‐type (WT; 145 bp) and mutant POLG (*Polg* ^mut^; 109 bp) alleles in e18.5 fetuses (Figure S1A). Hierarchical clustering of differentially expressed genes (DEGs) demonstrated a clear separation between WT and mutator, indicating a consistent genotype dependent transcriptional signature across biological replicates (Figure 1B). Consistently, DEG analysis identified a broad transcriptional shift between WT and *Polg* ^mut^ tissues, as illustrated by the principal component analysis (PCA) and volcano plot (Figure 1C,D), which revealed a substantial number of significantly upregulated and downregulated genes across the transcriptome. To gain functional insight into the transcriptional changes observed, we performed Hallmark gene set enrichment analysis (GSEA). As shown in Figure 1E, multiple metabolic pathways were significantly downregulated in POLG mutation, including oxidative phosphorylation (OXPHOS), fatty acid metabolism, adipogenesis, and myogenesis. In contrast, inflammatory response pathways were significantly enriched in the mutation, indicating a shift toward a pro‐inflammatory transcriptional state. Consistent with GSEA data, enrichment plots demonstrated strong negative enrichment of OXPHOS (NES = −2.638, FDR < 0.001) and fatty acid metabolism (NES = −2.001, FDR < 0.001), alongside positive enrichment of inflammatory response (NES = 1.670, FDR = 0.001) (Figure 1F). Gene ontology (GO)‐based GSEA and BP enrichment further supported suppression of mitochondrial respiration and ATP synthesis processes (Figure S1B–E).

To further investigate coordinated gene expression programs, we performed weighted gene co‐expression network analysis (WGCNA). Hierarchical clustering of genes, based on topological overlap, identified multiple distinct co‐expression modules (Figure 1G and Figure S2A), indicating structured transcriptional organization within the dataset. Correlation analysis between module eigengenes and genotype revealed several modules associated with the mutation, with the Module 19 exhibiting the strongest positive correlation (r = 0.93, *p* = 0.007). Module 17, Module 2, and Module 23 also showed moderate positive associations, whereas other Module 31, Module 21, and Module 12 were negatively correlated, suggesting the presence of both activated and suppressed transcriptional programs linked to genotype (Figure 1H). To validate the relationship between Module 19 and genotype, we examined module eigengene expression across groups. As shown in Figure 1I, Module 19 eigengene levels were markedly elevated in the mutation compared with WT, confirming that this module is strongly activated in the mutant condition. Functional characterization of Module 19 demonstrated enrichment of pathways, related to protein localization, secretion, and membrane trafficking (Figure S2B). In addition, hub gene analysis identified highly connected genes within the module that were strongly associated with genotype (Figure S2C, D), supporting the central role of this network in the observed transcriptional phenotype. Together, we demonstrate that RNA‐seq profiling reveals a coordinated transcriptional change characterized by suppressed mitochondrial metabolic programs and enhanced inflammatory signaling, which converge on a genotype‐associated co‐expression module driving the mutant phenotype.

### 3.2 POLG Mutation Impairs Fetal Bone Formation and Resorption

**FIGURE 2:**
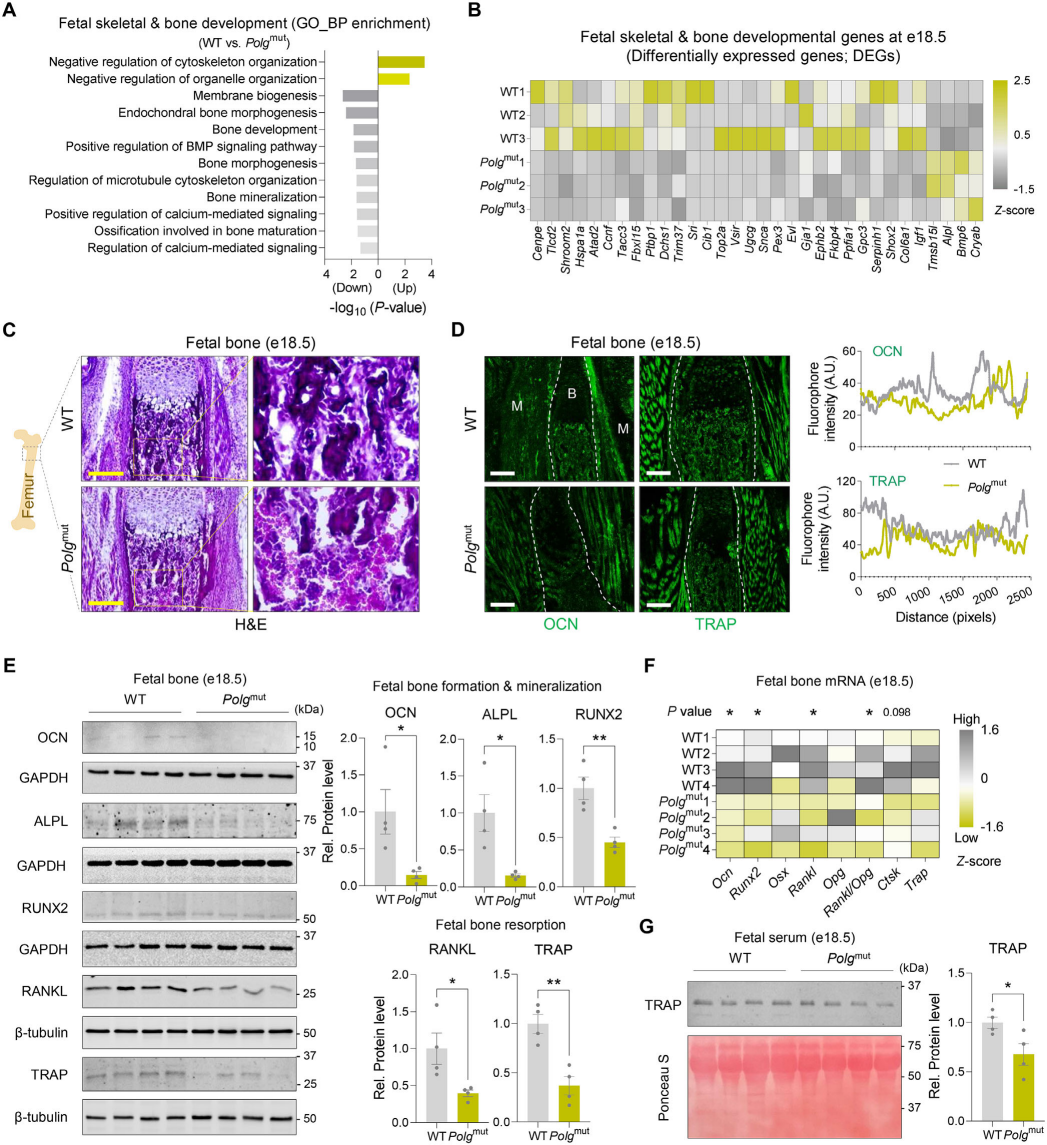
POLG mutation impairs fetal bone development and osteogenesis at e18.5. (A) Gene ontology (GO) biological process enrichment analysis of DEGs in fetal musculoskeletal tissues from WT and POLG mutant fetuses at e18.5. Selected bone‐related pathways are shown. (B) Heatmap of selected DEGs associated with fetal musculoskeletal development at e18.5. Each column represents an individual sample and each row represents a gene. Relative expression levels are shown as *Z*‐scores. (C) Representative images of H&E staining of fetal femur region at e18.5 from WT and POLG mutation. (D) Representative immunofluorescence images of OCN and TRAP staining in the fetal femur region at e18.5, with corresponding representative fluorescence intensity profiles from the displayed images. WT and *Polg* ^mut^ color annotations are indicated in the intensity profiles. Scale bar = 400 µm. (E) Cropped western blot images and relation protein levels of fetal bone formation & mineralization and bone resorption markers in e18.5 fetal bone (*n* = 4/group). (F) Relative mRNA expression in relation to fetal osteogenesis in e18.5 fetal bone (*n* = 4/group). (G) Cropped western blot images of bone resorption marker (TRAP) in serum (*n* = 4/group). Mean ± s.e.m., and each dot represents one litter. ^*^ *p* < 0.05, and ^**^ *p* < 0.01 in WT vs. *Polg* ^mut^ by two‐sided *p* values by Student *t*‐test (E–G).

Fetal muscle development occurs in concomitant to bone and tendon development [36], indicating a strong development and functional interaction between muscle and bone. GO enrichment analysis of the DEGs revealed that POLG mutation significantly perturbed developmental pathways associated with skeletal and muscular growth (Figure 2A). Downregulated pathways were strongly enriched for processes involved in cytoskeletal organization, supramolecular fiber organization, and organelle organization, together with biological programs regulating muscle cell differentiation, bone morphogenesis, bone mineralization, and endochondral ossification, suggesting impaired structural and developmental maturation of fetal musculoskeletal tissues (Figure 2B). Histological analysis revealed altered bone morphology in POLG mutant fetuses. Histological and immunofluorescence (IF) analyses showed that POLG mutation was associated with altered bone tissue characteristics in e18.5 fetal hindlimb. H&E staining revealed differences in the bone region architecture between WT and POLG mutation (Figure 2C). Consistently, representative osteocalcin (OCN) and tartrate‐resistant acid phosphatase (TRAP) immunofluorescence images showed reduced staining intensity in the fetal femur region of *Polg* ^mut^ fetuses compared with WT littermates (Figure 2D). Similarly, POLG mutation significantly reduced mRNA expression of key osteogenic and bone mineralization markers, including *Ocn* and *Runx2*, as well as bone resorption‐related markers such as *Rankl* and *Rankl*/*Opg* ratio, which is a primary determinant of bone remodeling balance (Figure 2F). At the protein level, markers associated with osteoblast activity and bone mineralization were also markedly reduced. Specifically, protein levels of OCN, alkaline phosphatase (ALPL), and the master transcription factor RUNX2, which are essential regulators of osteoblast differentiation and skeletal development, were significantly downregulated in fetal bone from *Polg* ^mut^ mice (Figure 2E). In addition to impaired osteoblast‐related signaling, indicators of osteoclast mediated bone resorption were also altered. Circulating levels of TRAP in fetal serum were significantly reduced in *Polg* ^mut^ mice (Figure 1G), together with decreased RANKL and TRAP protein expression in fetal bone, suggesting reduced osteoclast activity and impaired bone developmental capacity. Collectively, these findings demonstrate that POLG mutation disrupts fetal bone homeostasis by impairing both osteoblast‐driven bone formation and osteoclast‐mediated bone resorption, ultimately leading to defective fetal bone modeling. Together, these data suggest that POLG mutation is associated with decreased osteogenic and bone remodeling signals in the mutated fetal bone at e18.5.

### 3.3 PolG Mutagenesis Impedes Fetal Muscle Development

Extensive evidence supports the coordinated development and functional interplay between skeletal muscle and bone during fetal growth [37, 38, 39]. Histological examination of fetal skeletal muscle at e18.5 showed smaller myofiber size in *Polg* ^mut^ fetuses compared with WT controls, and quantitative analysis confirmed a significant reduction in mean myofiber cross‐sectional area (CSA) in *Polg* ^mut^ fetal muscle (Figure S3A). These findings indicate impaired fetal myofiber growth under POLG mutation. Consistently, POLG mutation significantly decreased the expression of key myogenic regulatory genes involved in skeletal muscle development and differentiation, including *Pax7*, *Myod1*, and *Myog* (Figure S3B). At the signaling level, POLG mutation suppressed anabolic pathways that regulate muscle hypertrophy. Specifically, phosphorylation of Akt at Thr308, mTOR at Ser2448, and the downstream effector P70S6K at Thr389 was markedly reduced in fetal muscle from *Polg* ^mut^ mice, indicating impaired activation of the Akt‐mTOR‐P70S6K signaling axis that controls protein synthesis and muscle growth (Figure S3C, D). Because mitochondrial function is essential for supporting skeletal muscle growth and differentiation [40], we next examined mitochondrial biogenesis in fetal muscle. POLG mutation significantly reduced the expression of mitochondrial biogenesis related genes, including *Pgc1*α and *Tfam*, and showed a decreasing trend for additional regulators such as *Pparg* and *Nrf1* (Figure S3E). In agreement with these transcriptional changes, protein levels of key mitochondrial regulators PGC‐1α, TFAM, and VDAC were markedly decreased in *Polg* ^mut^ fetal muscle (Figure S3F). Together, these findings demonstrate that POLG mutation disrupts fetal skeletal muscle development by suppressing myogenic gene programs, inhibiting anabolic hypertrophic signaling, and impairing mitochondrial biogenesis.

### 3.4 Maternal Exercise Rescues Fetal Osteogenesis Impaired Due to PolG Mutagenesis

**FIGURE 3:**
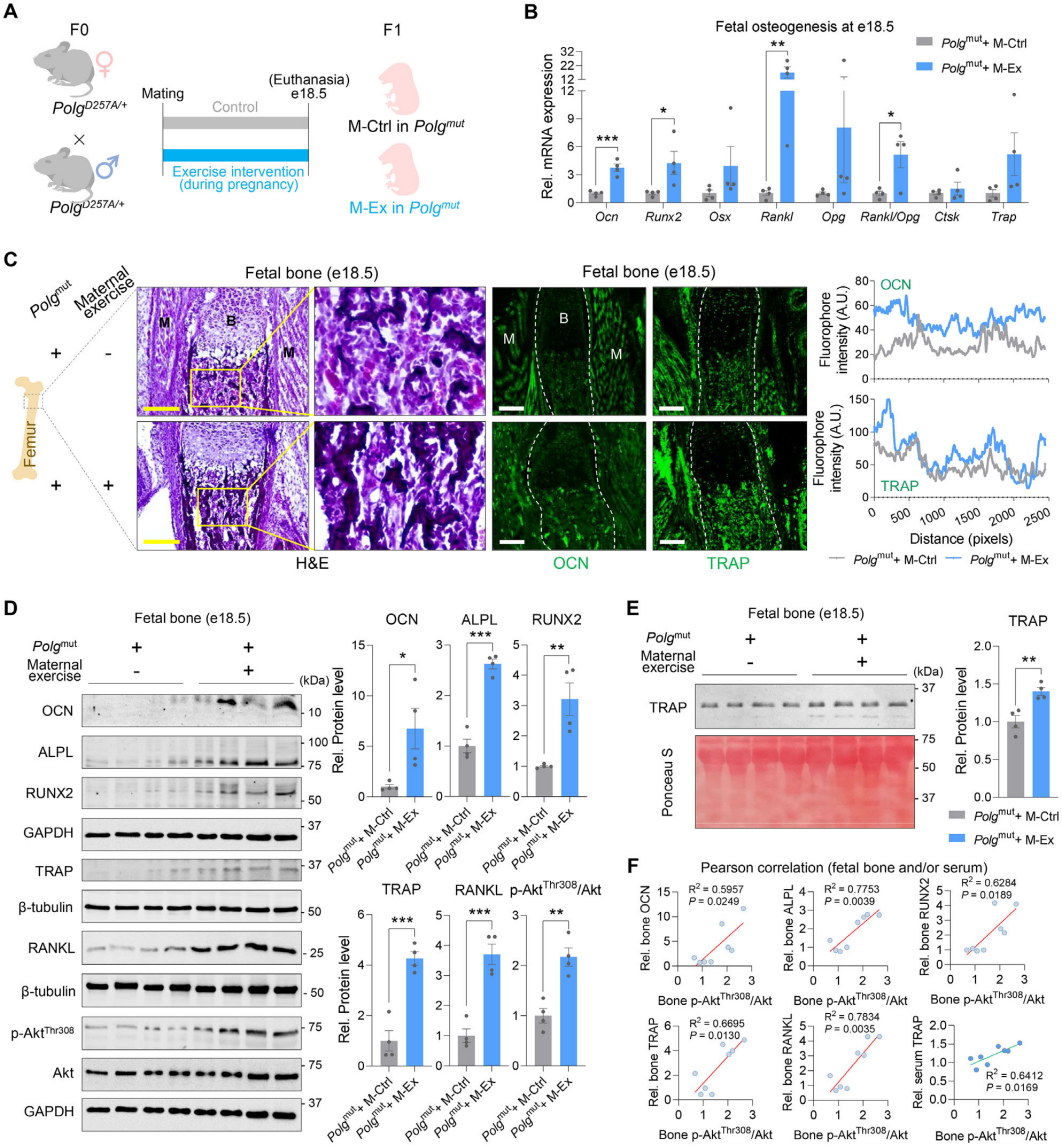
Maternal exercise restores fetal bone osteogenesis and homeostasis in POLG mutagenesis. (A) Research design to determine the effect of maternal exercise (ME) and POLG mutation on fetal bone homeostasis. (B) Relative mRNA expression of fetal osteogenesis‐related genes in fetal bone at e18.5 following maternal exercise (*n* = 4/group). (C) Representative H&E staining and immunofluorescence images of fetal femur region at e18.5 from *Polg* ^mut^ + M‐Ctrl and *Polg* ^mut^ + M‐Ex groups. OCN staining was used as a marker of osteoblast related bone formation, and TRAP staining was used to assess bone resorption. Fluorescence intensity was shown across the indicated region (M, muscle; B, bone; scale bar = 400 µm). (D) Cropped western blot images and relative protein levels of osteogenesis and Akt pathway in e18.5 fetal bone following ME (*n* = 4/group). (E) Cropped western blot images of circulating TRAP levels (*n* = 4/group). (F) Pearson correlations between Akt phosphorylation and osteogenic markers in fetal bone and serum (*n* = 4/group). Mean ± s.e.m., and each dot represents one litter. ^*^ *p* < 0.05, ^**^ *p* < 0.01, and ^***^ *p* < 0.001in *Polg* ^mut^+M‐Ctrl vs. *Polg* ^mut^+M‐Ex by two‐sided *p* values by Student *t*‐test (B‐E) and Pearson correlations (F).

ME promotes fetal muscle development and regulates oxidative metabolism [9, 10]. Considering the close relationship between muscle and bone development and homeostasis [41], we implemented an exercise intervention during pregnancy to determine whether ME also improves fetal bone development in *Polg* ^mut^ mice (Figure 3A) [32]. Histological H&E staining and OCN and TRAP IF analyses showed that ME partially restored the reduction of osteoblast and osteoclast activity, indicating increased bone turnover (Figure 3C). Consistently, ME significantly increased the mRNA expression of genes involved in osteogenesis and bone development in fetal bone at e18.5, including *Ocn* and *Runx2*, as well as genes related to bone resorption and formation such as *Rankl*, *Rankl*/*Opg* ratio (Figure 3B). In addition, protein analyses showed that ME increased osteoblast‐associated markers in fetal bone. Of note, protein levels of OCN, ALPL, and RUNX2, which regulate osteoblast differentiation and bone mineralization, were significantly elevated in *Polg* ^mut^ ME group compared with *Polg* ^mut^ controls (Figure 3D). Furthermore, markers associated with osteoclast activity were also increased. ME elevated RANKL and TRAP protein levels in fetal bone (Figure 3D), and circulating TRAP levels in fetal serum were significantly higher following ME (Figure 3E), indicating increased osteoclast activity. Mechanistically, these effects were accompanied by increased activation of the Akt signaling pathway, as shown by elevated phosphorylation of Akt at Thr308 in fetal bone (Figure 3D). Moreover, Akt activation was positively correlated with osteogenic and osteoclast markers, including OCN, ALPL, RUNX2, RANKL, and TRAP, in fetal bone and/or serum (Figure 3F). Together, these findings demonstrate that ME restores fetal bone homeostasis in *Polg* ^mut^ mice by enhancing both osteoblast‐ and osteoclast‐mediated bone formation and developmental balance.

### 3.5 Apelin‐APJ Signaling Axis is Associated with Fetal Osteogenesis

**FIGURE 4:**
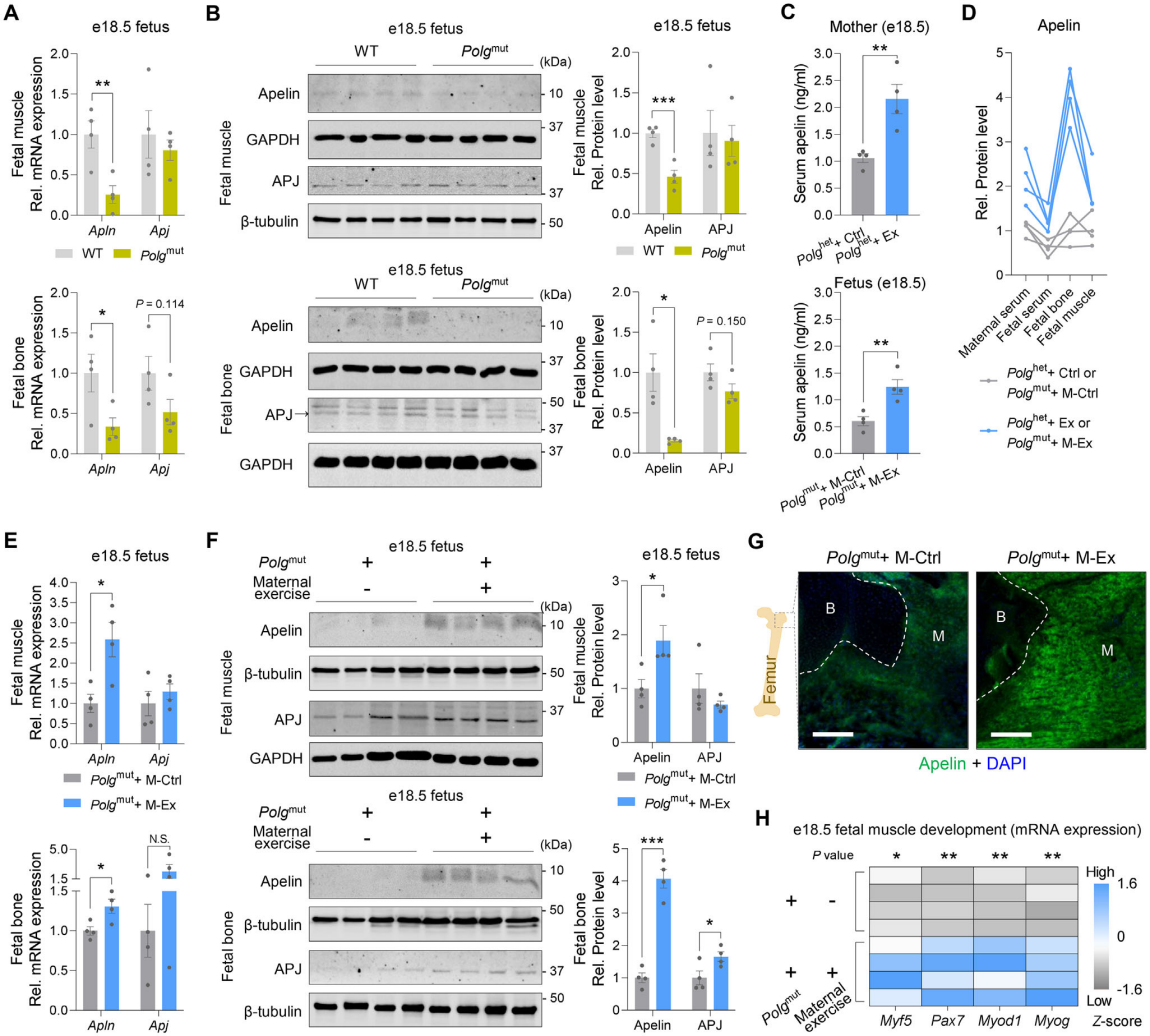
Maternal exercise activates skeletal muscle apelin expression for muscle‐bone interaction. (A) Relative gene expression of apelin signaling (*Apln* and *Apj*) in muscle (up) and bone (down) of WT and POLG mutation (*n* = 4/group). (B) Cropped western blot images and relative protein levels of Apelin and APJ in fetal muscle (up) and bone (down) (*n* = 4/group). (C) Maternal and fetal serum apelin levels in the POLG mutation pregnancy model. Maternal serum was compared between *Polg* heterozygous control and exercised dams, and fetal serum was compared between *Polg* ^mut^ fetuses from maternal control and maternal exercise pregnancies (*n* = 4/group). (D) Relative apelin protein levels across maternal serum, fetal serum, fetal bone, and fetal muscle in *Polg* ^mut^+M‐Ctrl and *Polg* ^mut^+M‐Ex groups (*n* = 4/group). (E) Relative gene expression of apelin signaling (*Apln* and *Apj*) in muscle (up) and bone (down) of POLG mutation following maternal exercise (*n* = 4/group). (F) Cropped western blot images and relative protein levels of Apelin and APJ in muscle (up) and bone (down) (*n* = 4/group). (G) Representative IF images of apelin staining (scale bar = 100 µm; B, bone; M, muscle). (H) Relative mRNA expression of myogenic genes in fetal muscle (*n* = 4/group). Mean ± s.e.m., and each dot represents one litter. ^*^ *p* < 0.05, ^**^ *p* < 0.01, and _***_ *p* < 0.001in WT vs. *Polg* ^mut^ or *Polg* ^mut^+M‐Ctrl vs. *Polg* ^mut^+M‐Ex by two‐sided *p* values by Student *t*‐test (A‐H).

It has been shown that apelin mimics exercise‐induced beneficial effects on fetal muscle development and its mitochondrial metabolism [9]. To examine whether the apelin and its receptor APJ pathway is associated with fetal bone development, we first utilized the POLG mutation model and examined apelin expression across fetal musculoskeletal tissues. POLG mutation altered apelin expression in fetal muscle and bone (Figure 4A,B). In the POLG mutation pregnancy model, ME increased circulating apelin levels in *Polg* heterozygous pregnant dams and in *Polg* ^mut^ fetuses compared with their respective maternal sedentary control groups (Figure 4C,D). ME also increased apelin abundance across maternal and fetal circulation and tissues, including maternal serum and fetal serum, muscle, and bone (Figure 4D–F). Notably, fetal bone itself showed detectable apelin expression under ME, suggesting that a bone‐local apelin response may contribute to fetal osteogenic regulation together with circulating, placental, and muscle‐associated apelin signals. In contrast to the changes in apelin abundance, APJ expression in fetal muscle and bone was relatively less responsive to POLG mutation and ME (Figure 4B,F), suggesting that apelin availability may represent the more prominently regulated component of the apelin‐APJ pathway in this mechanism. Because placenta represents as a key maternal‐fetal mediator, placental regulation may also contribute to the elevated fetal circulating apelin observed after ME [33]. Consistent with the previous study, we showed that ME enhances apelin expression in e18.5 POLG mutated placenta, decreased due to POLG mutation, especially in the labyrinth zone, the location where the maternal‐fetal interface occurs (Figure S4A–D). Moreover, immunofluorescence analysis revealed increased apelin immunoreactivity in skeletal muscle adjacent to developing bone in the M‐Ex group (Figure 4G), supporting a possible muscle‐to‐bone paracrine contribution. Together with the detectable apelin expression observed in fetal bone, these findings suggest that ME‐induced apelin signaling may involve both local fetal bone responses and inter‐tissue communication within the fetal musculoskeletal compartment. Consist with this observation, ME increased the expression of genes associated with fetal muscle development, including *Myf5*, *Pax7*, *Myod1*, and *Myog*, even in the presence of POLG mutation (Figure 4H).

**FIGURE 5:**
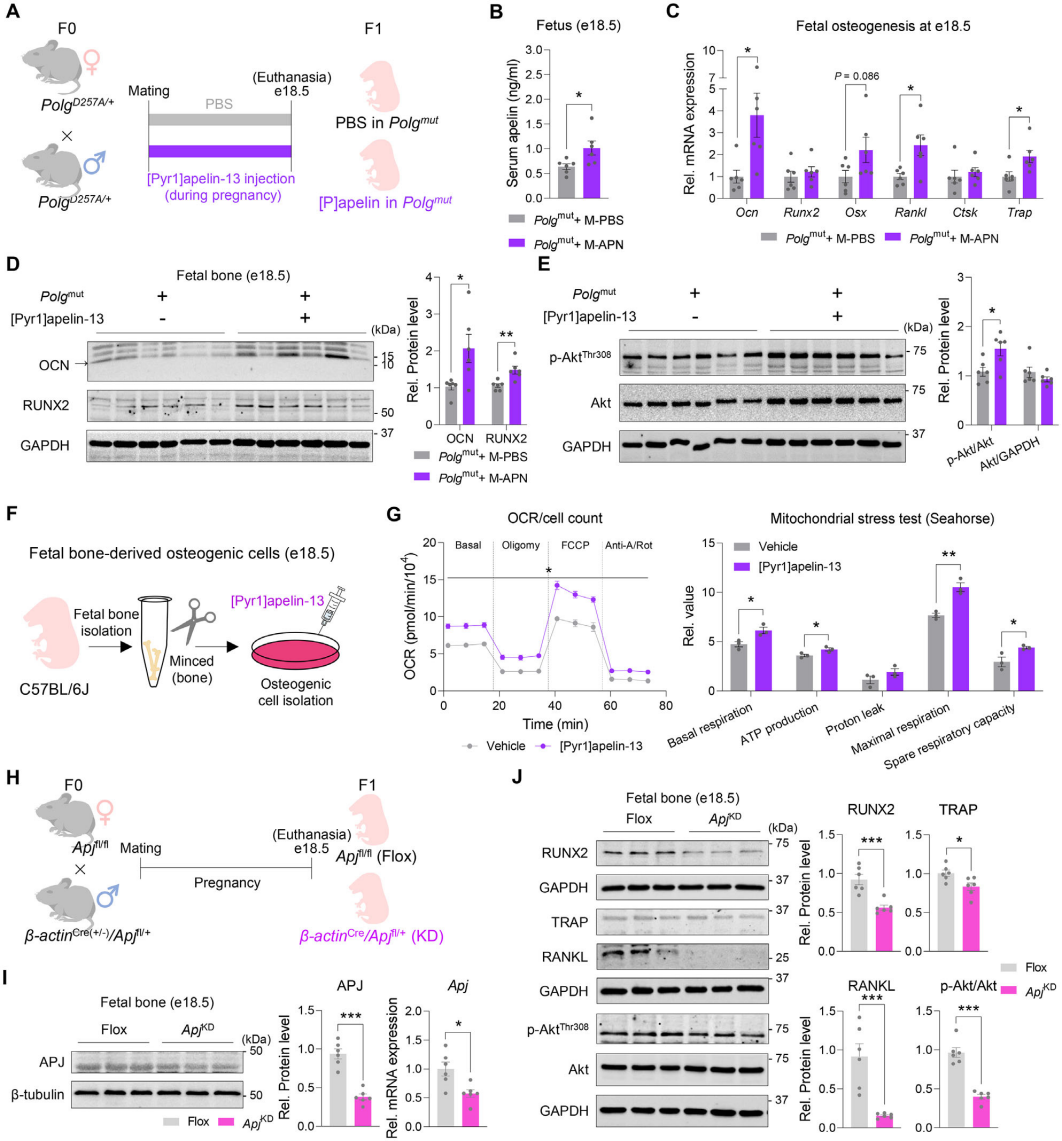
Apelin‐APJ axis regulates bone formation in POLG mutation. (A) Research design to investigate the impact of apelin administration during pregnancy in POLG mutated fetal bone at e18.5. (B) Circulating apelin levels of fetuses with maternal PBS injection (M‐PBS) or maternal apelin administration (M‐APN; n = 6/group). (C) mRNA expression of fetal osteogenesis in M‐PBS or M‐APN administration during pregnancy (*n* = 6). (D,E,) Cropped western blot analysis of fetal osteogenesis (D) and Akt pathway (E) in e18.5 fetal bone following apelin administration during pregnancy (*n* = 6). (F) Isolation of fetal bone‐derived osteogenic cells from WT pregnant mice with treatment of either 100 nm [Pyr1]apelin‐13 or vehicle. (G) Seahorse mitochondrial stress test after either [Pyr1]apelin‐13 or vehicle treatment in fetal bone‐derived osteogenic cells (*n* = 3). (H, I) Experimental design for obtaining APJ deficient fetal bone (H) and their APJ expression (I). (J) Cropped western blot analysis of fetal osteoblast and osteoclast, as well as Akt pathway in APJ knockdown (KD) fetal bone (*n* = 6/group). Mean ± s.e.m., and each dot represents one litter. ^*^ *p* < 0.05, ^**^ *p* < 0.01, and ^***^ *p* < 0.001 in *Polg* ^mut^+M‐PBS vs. *Polg* ^mut^+M‐APN or Flox vs. *Apj* ^KD^ by two‐sided *p* values by Student *t*‐test (B‐J).

To directly determine the effect of apelin administration during pregnancy, we conducted an additional animal study using [Pyr1]apelin‐13 administration (Figure 5A). Consistent with our previous study showing that maternal apelin administration during pregnancy increases fetal circulating apelin levels [33], apelin treatment during pregnancy significantly increased circulating apelin levels in *Polg* ^mut^ fetuses and increased the expression of fetal bone osteogenic and remodeling genes, including *Ocn*, *Rankl*, and *Trap* (Figure 5B,C). Consistently, protein levels of osteogenic markers OCN and RUNX2 were markedly increased following apelin administration (Figure 5D). These effects were accompanied by increased Akt phosphorylation (Thr308) in fetal bone, indicating activation of the Akt signaling pathway (Figure 5D). To investigate mitochondrial cellular function, we isolated bone‐derived osteogenic cells from e18.5 WT fetal skeletal tissues and treated them with either vehicle or 100 nm [Pyr1]apelin‐13 for 24 h. Seahorse extracellular flux analysis showed that apelin significantly increased basal respiration, ATP‐linked respiration, maximal respiration, and spare respiratory capacity, whereas proton leak was not significantly changed (Figure 5F,G), indicating that apelin enhances mitochondrial respiratory function and bioenergetic capacity in fetal bone‐derived osteogenic cells.

To further determine whether APJ is involved in fetal musculoskeletal development, we performed an additional experiment using *Apj*‐deficient fetuses at e18.5 (Figure 5H). APJ mRNA expression and protein levels were markedly reduced in *Apj* ^KD^ fetal bone compared with Flox controls (Figure 5I). Importantly, *Apj* ^KD^ fetuses showed reduced protein levels of osteogenic and osteoclast‐related markers, including RUNX2, RANKL, and TRAP, in fetal bone (Figure 5J). We further observed downregulation of the Akt phosphorylation in *Apj* ^KD^ fetal bone, consistent with decreased APJ‐associated downstream signaling (Figure 5J). Because the *Apj* ^KD^ model was generated using *β‐actin* ^Cre^, we next examined whether APJ reduction also occurred in fetal muscle and whether fetal myogenic gene expression was affected. APJ gene expression and protein level were decreased in *Apj* ^KD^ fetal muscle (Figure S5A,B). In addition, *Apj* ^KD^ reduced the mRNA expression of fetal myogenic markers, including *Myf5*, *Myod1*, and *Myog* (Figure S5C). Together, these findings suggest that APJ deficiency affects fetal muscle development as well as fetal bone osteogenic signaling. Since *β‐actin* ^Cre^ broadly reduces APJ expression, the reduced osteogenic markers in *Apj* ^KD^ fetal bone may reflect both bone APJ signaling and broader musculoskeletal changes involving fetal muscle.

### 3.6 ME‐Induced Apelin Signaling Rescues Fetal Bone Mitochondrial Biogenesis impaired Due to PolG Mutations

ME induces mitochondrial biogenesis in fetal tissues, including brown fat and muscle [9, 33], but its role in fetal bone mitochondrial metabolism has not been well characterized. To determine whether ME affects fetal bone mitochondrial metabolism, we analyzed fetal bone from POLG mutation model with ME intervention. ME reversed the reductions in mtDNA copy number and mitochondrial biogenesis‐related gene expression caused by POLG mutation in fetal bone (Figure S6A–F). Specifically, ME increased gene expression and protein levels of key mitochondrial regulators, including PGC‐1α and TFAM (Figure S6E, F), which were markedly suppressed by POLG mutagenesis (Figure S6A–C). As an exercise‐mimetic hormone, apelin administration during pregnancy similarly increased mtDNA copy number and PGC‐1α expression, a master regulator of mitochondrial biogenesis and energy metabolism (Figure S6G–I). Interestingly, we found that PGC‐1α expression is regulated by epigenetic modifications. GO analysis of the e18.5 fetal musculoskeletal RNA‐seq dataset revealed that pathways associated with epigenetic regulation and histone demethylation were significantly altered in POLG mutation (Figure S7A). Because previous studies demonstrated that both ME and apelin promote DNA demethylation of the *Pgc1a* promoter in fetal tissues [9, 33], we examined DNA methylation enrichment in the CpG island of the *Pgc1a* promoter previously characterized in our studies (Figure S7B). POLG mutation significantly increased the enrichment of 5‐methylcytosine (5mC), a DNA methylation mark associated with transcriptional repression, in the *Pgc1a* promoter of e18.5 fetal bone (Figure S7C). In contrast, ME and apelin administration during pregnancy reduced 5mC enrichment at the *Pgc1a* promoter region in fetal bone (Figure S7D, E). These changes were associated with increased *Pgc1a* expression, suggesting that reduced promoter 5mC enrichment may contribute to the regulation of *Pgc1a* expression in this model. Together, these results demonstrate that ME‐induced apelin signaling restores mitochondrial biogenesis in fetal bone by relieving epigenetic suppression of *Pgc1a* caused by POLG mutation.

### 3.7 Fetal Osteogenesis is Mediated by ATF4‐RUNX2 Coregulation

**FIGURE 6:**
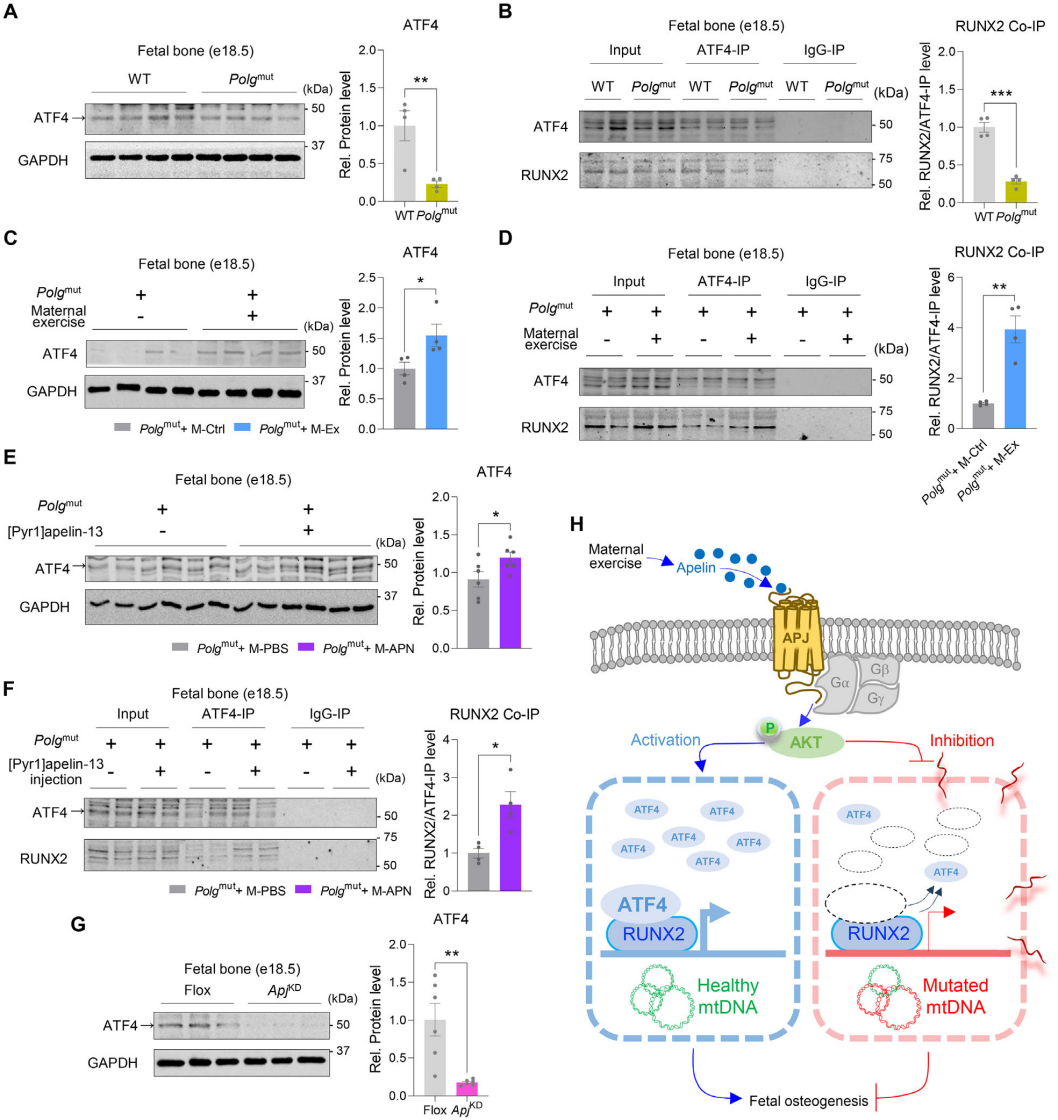
ME‐apelin signaling axis‐dependent co‐regulation of ATF4‐RUNX2 complex in POLG mutation. (A,B) Cropped western blot analyses of ATF4 protein levels (A; normalized by GAPDH) and the co‐regulation between ATF4 and RUNX2 in POLG mutation (B; n = 4/group). (C,D) Cropped western blot images of ATF4 protein levels (C; normalized by GAPDH) and the co‐regulation between ATF4 and RUNX2 in POLG mutation following ME (D; n = 4/group). (E,F) Cropped western blot images of ATF4 protein levels (E; normalized by GAPDH; n = 6/group) and the co‐regulation between ATF4 and RUNX2 in POLG mutation following apelin administration during pregnancy (F; n = 4/group). Interpretation of Co‐IP signals takes into account differences in input protein levels. (G) Cropped western blot analysis of ATF4 in APJ knockdown fetal bone (*n* = 6/group). (H) Proposed schematic diagram in ME‐apelin‐APJ axis in regulation of ATF4‐RUNX2 co‐regulation, leading to enhanced fetal osteogenesis and formation. Mean ± s.e.m., and each dot represents one litter. ^*^ *p* < 0.05 and ^**^ *p* < 0.01, in WT vs. *Polg* ^mut^, *Polg* ^mut^+M‐Ctrl vs. *Polg* ^mut^+M‐Ex, or *Polg* ^mut^+M‐PBS vs. *Polg* ^mut^+M‐APN by two‐sided *p* values by Student *t*‐test (A‐G).

POLG mutation significantly reduced the expression of activating transcription factor 4 (ATF4) in fetal bone, whereas ME restored ATF4 protein levels (Figure 6A,C). We further investigated whether ATF4 cooperates with RUNX2 to regulate fetal osteogenic signaling. Co‐immunoprecipitation (Co‐IP) analysis showed that POLG mutation markedly reduced the co‐regulation and association between ATF4 and RUNX2 in fetal bone, indicating impaired formation of the ATF4‐RUNX2 transcriptional complex (Figure 6B). In contrast, ME increased the association between ATF4 and RUNX2, suggesting restoration of their transcriptional coregulatory activity (Figure 6D). To determine whether apelin mediates this effect, we administered the exercise mimetic hormone [Pyr1]apelin‐13 during pregnancy. Apelin administration increased ATF4 protein expression and enhanced the co‐regulation between ATF4 and RUNX2 in fetal bone (Figure 6E,F). To further confirm the role of the apelin receptor APJ, we examined fetal bone from *Apj*‐deficient mice. APJ knockdown markedly reduced ATF4 protein expression compared with Flox controls (Figure 6G), supporting an association between APJ signaling and ATF4 abundance in fetal bone. Together, these findings suggest that ME and apelin are associated with enhanced ATF4 abundance and ATF4‐RUNX2 co‐immunoprecipitation signal in fetal bone, supporting a potential apelin‐APJ‐ATF4‐RUNX2 regulatory pathway in fetal osteogenic responses (Figure 6H).

## 4 Discussion

**FIGURE 7:**
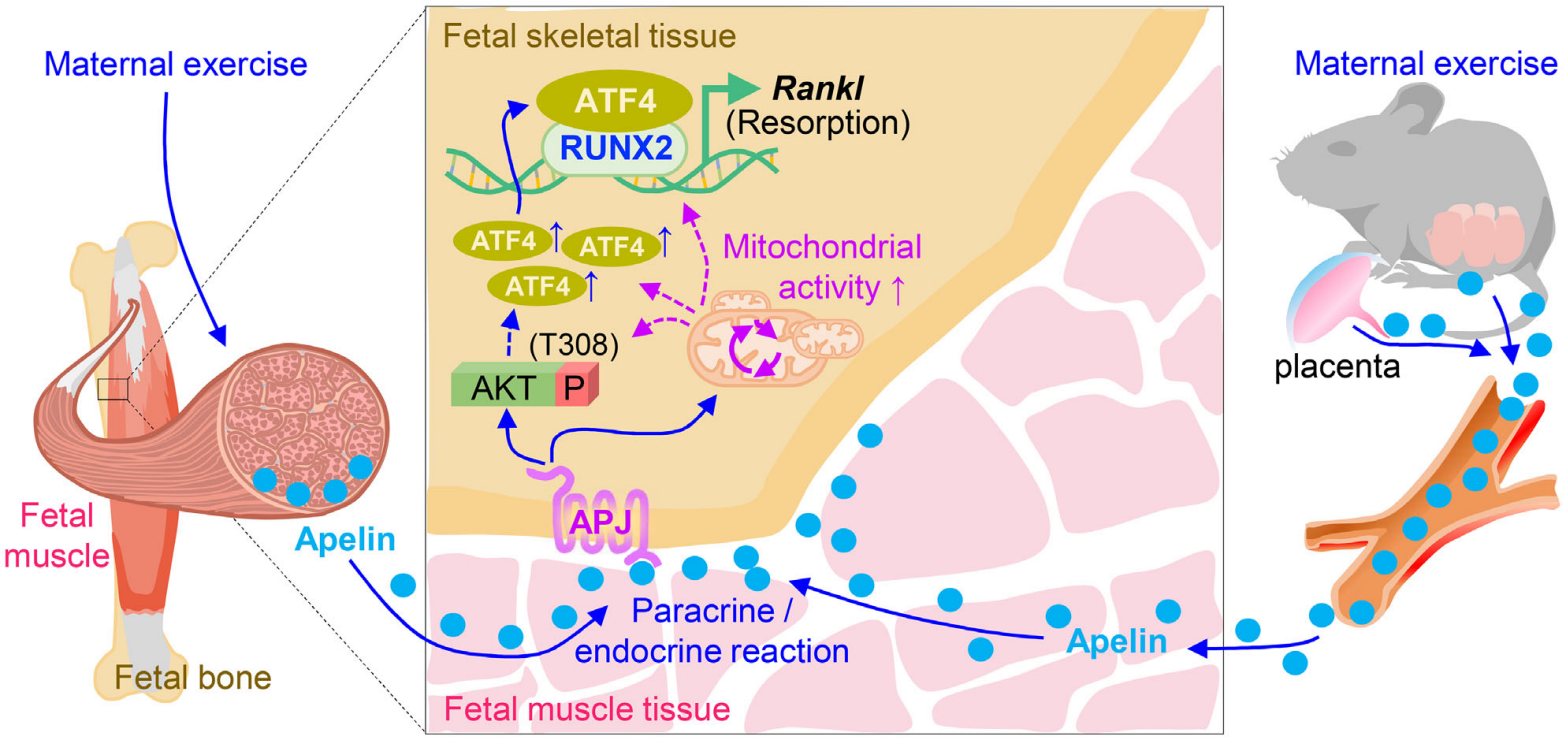
Potential schematic diagram of proposed pathway. Proposed model for maternal exercise‐associated apelin‐APJ signaling in fetal musculoskeletal development under POLG mutation. Maternal exercise (ME) increases apelin abundance across maternal and fetal circulation and tissues, including maternal serum, fetal serum, placenta, fetal muscle, and fetal bone. Increased apelin availability may activate APJ‐associated signaling within fetal musculoskeletal tissues, leading to changes in Akt phosphorylation, mitochondrial biogenesis‐related markers, and ATF4‐RUNX2 association. These responses are associated with improved fetal osteogenic and bone remodeling markers.

Mitochondrial POLG point mutations lead to mitochondrial DNA depletion and impair mitochondrial biogenesis [42, 43], adversely affecting muscle development [44] and contributing to low bone mass and malformations [45, 46]. However, the underlying mechanisms and therapeutic potential remain incompletely understood. In this study, we show that ME improves fetal osteogenic and bone remodeling markers impaired due to POLG mutation in association with enhanced apelin signaling. POLG mutation disrupted fetal muscle and bone development by downregulating pathways involved in cytoskeletal organization, fiber organization, and mitochondrial metabolism. In contrast, ME increased apelin abundance across multiple maternal and fetal circulation and tissues, including maternal serum, fetal serum, fetal muscle, placenta, and fetal bone. Importantly, fetal bone itself showed detectable apelin expression under ME, suggesting that fetal bone may also serve as an active apelin‐expressing tissue during osteogenic regulation. By comparison, APJ expression in fetal muscle and bone was relatively stable, suggesting that ME may regulate this pathway mainly by increasing apelin availability rather than by markedly changing receptor abundance. Thus, our data support a model in which ME‐induced apelin signaling may regulate fetal osteogenesis through both local fetal bone responses and inter‐tissue communication within the maternal‐fetal musculoskeletal axis. We further show that apelin signaling increased the expression of ATF4 and enhanced co‐regulation between ATF4 and RUNX2 reduced due to POLG mutation. This likely promotes RANKL expression, contributing to bone formation and developmental balance. All data provide new insights into the developmental origins of fetal bone formation and homeostasis by apelin‐APJ signaling across maternal and fetal compartments and suggest potential therapeutic targets for fighting mitochondrial disorder‐related bone developmental retardation (Figure 7).

Functional POLG activity is required for healthy embryogenesis [12]. Mutations in POLG lead to mtDNA mutations and loss of DNA proofreading capacity, disrupting mtDNA maintenance and impairing mitochondrial enzymatic function [42]. Specifically, mtDNA mutations in oocytes have been shown to cause developmental defects through failure of epigenetic reprogramming associated with abnormal DNA methylation [47]. Consistently, we previously demonstrated that obesity‐induced AMPK deficiency can induce oocyte mtDNA heteroplasmy [48]. In addition, our recent data show that POLG mutations trigger muscle atrophy and intramuscular fibrosis, exacerbated by maternal high‐fat diet (HFD)‐induced metabolic dysfunction [49]. Although POLG‐related disorders, including seizures, hypotonia, muscle weakness and dystrophy, and neuropathy, are well documented, the effects of POLG mutations on fetal bone formation by apelin signaling across maternal and fetal compartments including blood and musculoskeletal tissues remain unexplored. Using POLG mutant mice as a model of mitochondrial dysfunction, our study provides evidence that impaired mitochondrial metabolism disrupts fetal bone formation and homeostasis, highlighting its role in skeletal integrity. Although reduced 5mC enrichment at the *Pgc1a* promoter was associated with increased *Pgc1a* expression, the present study does not establish a causal epigenetic mechanism. Future studies directly manipulating DNA methylation at the *Pgc1a* promoter will be needed to determine whether this change drives *Pgc1a* induction in fetal bone.

Regular physical activity promotes optimal bone health by enhancing muscle strength and bone density through increased mechanical loading and osteogenic signaling [50]. Recent studies suggest that maternal exercise during pregnancy also positively influences early‐life bone development [50]. For example, a randomized controlled trial in humans showed that maternal exercise improves bone mineral acquisition in premature infants [51]. Interestingly, exercise has also been shown to counteract progeroid aging through systemic mitochondrial rejuvenation in mtDNA‐mutated models [52] and to improve metabolic function in the brains of POLG mutated mice [53]. Similarly, exercise has been shown to improve oocyte quality under POLG mutant conditions [54]. Despite these findings, the developmental impact of maternal exercise on fetal bone development has not been investigated.

Apelin is known to promote osteogenic differentiation in human bone marrow‐derived mesenchymal stem cells [22], likely through enhanced mitochondrial biogenesis and metabolic activity [20, 55]. The apelin‐APJ axis has been shown to enhance mitochondrial biogenesis and oxidative metabolism in various tissues, including skeletal muscle [20, 55] and brown fat [56]. In particular, our previous studies have shown that apelin administration during pregnancy boosts mitochondrial energy metabolism in the placenta [57], as well as in offspring muscle [9, 10], brown and beige fat [33], and the small intestine [58]. While numerous studies have examined the role of apelin in mitochondrial function, its role in regulating fetal bone formation and homeostasis, as well as interaction between muscle and bone has not been clearly defined. We have shown that maternal exercise increases circulating apelin levels in mother and fetus, as well as placenta as a mediator linking mother with her fetus [33]. Consistently, the present study showed ME increased circulating apelin levels in both maternal and fetal compartments at e18.5 even under POLG mutation, indicating that ME is associated with enhanced apelin signaling in maternal circulation, the placenta, and the fetal compartment. However, the relative contribution of maternal transfer, placental regulation, and local fetal apelin production remains to be determined. In particular, the increase of apelin signal observed in fetal bone suggests that fetal bone may not only respond to apelin derived from maternal circulation, placenta, or fetal muscle, but may also participate as a local apelin‐expressing tissue. Local bone apelin expression may, therefore, act together with circulating and muscle‐associated apelin signals to support fetal osteogenic regulation. Future studies using tissue‐specific *Apln* deletion in fetal muscle, placenta, or osteogenic lineage cells will be useful to define the relative contribution of each apelin source to fetal bone development.

The additional characterization of *Apj* ^KD^ fetal muscle further supports the broader role of APJ signaling in fetal musculoskeletal development. In *Apj* ^KD^ fetuses, APJ mRNA and protein levels were reduced in fetal muscle, accompanied by decreased expression of myogenic genes. These findings indicate that APJ deficiency affects fetal muscle developmental programs as well as fetal bone osteogenic markers. Therefore, the reduced osteogenic signaling observed in *Apj* ^KD^ fetal bone may be influenced by both local bone APJ signaling and altered fetal muscle development. Because *β‐actin* ^Cre^ broadly reduces APJ expression, future studies using tissue‐specific *Apj* deletion in osteogenic, osteoclast‐lineage, or skeletal muscle lineages will be needed to define the relative contribution of each tissue to fetal muscle‐bone development. In addition, future studies combining ME or maternal apelin administration with tissue‐specific *Apj* deletion in *Polg* mutant fetuses will be necessary to determine whether APJ is required for the ME‐ or apelin‐induced improvement of fetal osteogenesis under mitochondrial dysfunction.

The observed changes in Akt phosphorylation suggest that Akt‐associated signaling may be linked to apelin‐APJ‐related fetal osteogenic regulation. However, the central focus of this study is the maternal exercise‐induced apelin‐APJ axis, and the present data do not establish Akt as a required downstream mediator because direct Akt inhibition or genetic Akt loss‐of‐function was not performed. Future studies focused on the downstream signaling mechanisms of apelin‐APJ pathway will be needed to determine whether Akt activity is necessary for ME‐ or apelin‐induced fetal osteogenic regulation. Such studies will require experimental designs optimized for the pregnancy‐fetal bone model, including the timing of intervention, fetal exposure, tissue specificity, and potential developmental effects.

ATF4 is a key transcription factor involved in skeletal development. The muscle‐specific *Atf4* knockout mice exhibit premature aging phenotypes [59]. Furthermore, ATF4‐dependent transcriptional programs also mitigate mitochondrial dysfunction resulting from endoplasmic reticulum (ER) stress [60]. Our data indicated that ATF4 expression was significantly reduced in fetal bone under POLG mutation but was restored by ME. Notably, ME also enhanced the co‐regulation between ATF4 and RUNX2, a complex that likely regulates RANKL expression and contributes to bone homeostasis. Our findings suggest a potential change and mechanistic link in ATF4‐RUNX2 association and RANKL transcription in fetal bone in response to ME‐induced apelin‐APJ signaling axis.

Because the present study focused on fetal osteogenesis at e18.5, we did not determine whether the beneficial effects of ME persist into postnatal skeletal development. Thus, it remains unknown whether ME‐induced improvements in fetal osteogenic and bone remodeling markers translate into sustained postnatal gains in bone accrual, mineralization, or musculoskeletal function. Longitudinal studies will be required to determine the durability and functional relevance of these fetal skeletal changes after delivery.

In conclusion, this study advances our understanding of how ME‐induced apelin signaling regulates fetal bone formation and homeostasis during fetal development, particularly under conditions of POLG mutation‐induced mitochondrial dysfunction. While previous studies have addressed the impact of POLG mutation on skeletal muscle, its role in fetal bone metabolism and the potential therapeutic effects of exercise had not been elucidated. We show that POLG mutation impairs fetal bone morphogenesis, disrupts cytoskeletal integrity, and reduces fetal bone formation and development. The concurrent reduction in osteoblast‐ and osteoclast‐associated markers suggests reduced bone turnover and may also reflect delayed skeletal development. Importantly, we demonstrate that the ME‐apelin signaling axis improves bone homeostasis by enhancing ATF4‐RUNX2 transcriptional activity through APJ activation. These findings identify a novel therapeutic avenue for treating mitochondrial dysfunction‐induced skeletal disorders during fetal development.

## Author Contributions

S.A.C. designed, performed experiments, analyzed, interpreted the data, wrote the manuscript, and edited the manuscript. R.C.R. analyzed and interpreted the data and edited the manuscript. Z.J. performed sequencing data analysis and interpreted the data. C.S. analyzed and interpreted the data. M.D. and J.S.S. designed, interpreted the data, and edited the manuscript.

## Conflicts of Interest

The authors declare no conflict of interest.

## Supporting information




**Supporting File**: advs77029‐sup‐0001‐SuppMat.pdf.

## Data Availability

The data that support the findings of this study are available from the corresponding author upon reasonable request.

## References

[1] L. Bonewald , “Use it or Lose it to Age: A Review Of Bone And Muscle Communication,” Bone 120 (2019): 212, 10.1016/j.bone.2018.11.002. 30408611 PMC6360108

[2] R. Sheng , M. Cao , M. Song , et al., “Muscle‐Bone Crosstalk Via Endocrine Signals And Potential Targets For Osteosarcopenia‐Related Fracture,” Journal of Orthopaedic Translation 43 (2023): 36–46, 10.1016/j.jot.2023.09.007. 38021216 PMC10654153

[3] T. Bettis , B. J. Kim , and M. W. Hamrick , “Impact of muscle atrophy on bone metabolism and bone strength: Implications for muscle‐bone crosstalk With aging and disuse,” Osteoporosis International 29 (2018): 1713–1720, 10.1007/s00198-018-4570-1. 29777277 PMC7861141

[4] A. Torres‐Costoso , P. López‐Muñoz , V. Martínez‐Vizcaíno , C. Álvarez‐Bueno , and I. Cavero‐Redondo , “Association Between Muscular Strength and Bone Health From Children to Young Adults: A Systematic Review and Meta‐Analysis,” Sports Medicine 50 (2020): 1163–1190, 10.1007/s40279-020-01267-y. 32060751

[5] M. Locquet , C. Beaudart , N. Durieux , J. Y. Reginster , and O. Bruyère , “Relationship Between the Changes Over Time Of Bone Mass And Muscle Health In Children And Adults: A Systematic Review And Meta‐Analysis,” BMC Musculoskeletal Disorders 20 (2019): 429, 10.1186/s12891-019-2752-4. 31521141 PMC6745072

[6] S. E. McCormack , D. L. Cousminer , A. Chesi , et al., “Association Between Linear Growth and Bone Accrual in a Diverse Cohort of Children and Adolescents,” JAMA Pediatrics 171 (2017): 171769, 10.1001/jamapediatrics.2017.1769. PMC5632753 28672287

[7] S. Y. Jang , J. Park , S. Y. Ryu , and S. W. Choi , “Low Muscle Mass Is Associated With Osteoporosis: A Nationwide Population‐Based Study,” Maturitas 133 (2020): 54–59, 10.1016/j.maturitas.2020.01.003. 32005424

[8] S. Kim , C. W. Won , B. S. Kim , H. R. Choi , and M. Y. Moon , “The Association Between the Low Muscle Mass and Osteoporosis in Elderly Korean People,” Journal of Korean Medical Science 29 (2014): 995, 10.3346/jkms.2014.29.7.995. 25045234 PMC4101790

[9] J. S. Son , S. A. Chae , H. Wang , et al., “Maternal Inactivity Programs Skeletal Muscle Dysfunction in Offspring Mice by Attenuating Apelin Signaling and Mitochondrial Biogenesis,” Cell Reports 33 (2020): 108461, 10.1016/j.celrep.2020.108461. 33264618 PMC8137280

[10] J. S. Son , S. A. Chae , L. Zhao , et al., “Maternal Exercise Intergenerationally Drives Muscle‐Based Thermogenesis Via Activation Of Apelin‐AMPK Signaling,” EBioMedicine 76 (2022): 103842, 10.1016/j.ebiom.2022.103842. 35081489 PMC8790600

[11] A. Trifunovic , A. Wredenberg , M. Falkenberg , et al., “Premature Ageing In Mice Expressing Defective Mitochondrial DNA Polymerase,” Nature 429 (2004): 417–423, 10.1038/nature02517. 15164064

[12] N. Hance , M. I. Ekstrand , and A. Trifunovic , “Mitochondrial DNA Polymerase Gamma Is Essential For Mammalian Embryogenesis,” Human Molecular Genetics 14 (2005): 1775, 10.1093/hmg/ddi184. 15888483

[13] J. Suh and Y. S. Lee , “The Multifaceted Roles Of Mitochondria In Osteoblasts: From Energy Production To Mitochondrial‐Derived Vesicle Secretion,” Journal of Bone and Mineral Research 39 (2024): 1205–1214, 10.1093/jbmr/zjae088. 38907370 PMC11371665

[14] S. A. Mohammed , S. Ambrosini , T. Lüscher , F. Paneni , and S. Costantino , “Epigenetic Control Of Mitochondrial Function In The Vasculature,” Frontiers in Cardiovascular Medicine 7 (2020): 28, 10.3389/fcvm.2020.00028. 32195271 PMC7064473

[15] A. Castegna , V. Iacobazzi , and V. Infantino , “The Mitochondrial Side Of Epigenetics,” Physiological Genomics 47 (2015): 299–307, 10.1152/physiolgenomics.00096.2014. 26038395

[16] C. Zhang , Y. Meng , and J. Han , “Emerging Roles Of Mitochondrial Functions And Epigenetic Changes In The Modulation Of Stem Cell Fate,” Cellular and Molecular Life Sciences 81 (2024): 26, 10.1007/s00018-023-05070-6. 38212548 PMC11072137

[17] P. Yue , H. Jin , S. Xu , et al., “Apelin Decreases Lipolysis via Gq, Gi, and AMPK‐Dependent Mechanisms,” Endocrinology 152 (2011): 59–68, 10.1210/en.2010-0576. 21047945 PMC3033059

[18] J. S. Son , S. A. Chae , E. D. Testroet , M. Du , and H. P. Jun , “Exercise‐Induced Myokines: A Brief Review Of Controversial Issues Of This Decade,” Expert Review of Endocrinology & Metabolism 13 (2018): 51, 10.1080/17446651.2018.1416290. 30063442

[19] K. Higuchi , T. Masaki , K. Gotoh , et al., “Apelin, an APJ Receptor Ligand, Regulates Body Adiposity and Favors the Messenger Ribonucleic Acid Expression of Uncoupling Proteins in Mice,” Endocrinology 148 (2007): 2690–2697, 10.1210/en.2006-1270. 17347313

[20] C. Attané , C. Foussal , S. Le Gonidec , et al., “Apelin Treatment Increases Complete Fatty Acid Oxidation, Mitochondrial Oxidative Capacity, And Biogenesis In Muscle Of Insulin‐Resistant Mice,” Diabetes 61 (2012): 310, 10.2337/db11-0100. 22210322 PMC3266414

[21] Y. H. Wang , Y. Y. Wu , C. H. Tsai , et al., “High p53 and MAP1 Light Chain 3A co‐Expression Predicts Poor Prognosis in Patients With Esophageal Squamous Cell Carcinoma,” Molecular Medicine Reports 8 (2026): 41–46, 10.3892/mmr.2025.13751. 23632916

[22] K. Hang , C. Ye , J. Xu , et al., “Apelin Enhances The Osteogenic Differentiation Of Human Bone Marrow Mesenchymal Stem Cells Partly Through Wnt/β‐Catenin Signaling Pathway,” Stem Cell Research & Therapy 10 (2019): 189, 10.1186/s13287-019-1286-x. 31238979 PMC6593611

[23] Z. Zhao , K. Yan , Q. Guan , Q. Guo , and C. Zhao , “Mechanism and Physical Activities In Bone‐Skeletal Muscle Crosstalk,” Frontiers in Endocrinology 14 (2023): 1287972, 10.3389/fendo.2023.1287972. 38239981 PMC10795164

[24] J. Luo , W. Liu , F. Feng , and L. Chen , “Apelin/APJ system: A Novel Therapeutic Target For Locomotor System Diseases,” European Journal of Pharmacology 906 (2021): 174286, 10.1016/j.ejphar.2021.174286. 34174264

[25] X. Zhang , S. Yu , D. L. Galson , et al., “Activating Transcription factor 4 is Critical For Proliferation And Survival In Primary Bone Marrow Stromal Cells And Calvarial Osteoblasts,” Journal of Cellular Biochemistry 105 (2008): 885–895, 10.1002/jcb.21888. 18729081 PMC2704124

[26] Y. Xiao , X. Xie , Z. Chen , G. Yin , W. Kong , and J. Zhou , “Advances in the Roles of ATF4 in Osteoporosis,” Biomedicine & Pharmacotherapy 169 (2023): 115864, 10.1016/j.biopha.2023.115864. 37948991

[27] M. L. Sohaskey , Y. Jiang , J. J. Zhao , A. Mohr , F. Roemer , and R. M. Harland , “Osteopotentia Regulates Osteoblast Maturation, Bone Formation, And Skeletal Integrity In Mice,” Journal of Cell Biology 189 (2010): 511–525, 10.1083/jcb.201003006. 20440000 PMC2867309

[28] T. Komori , “Regulation of Osteoblast Differentiation by Runx2,” Advances in Experimental Medicine and Biology 658 (2010): 43, 10.1007/978-1-4419-1050-9_5. 19950014

[29] P. Ducy , R. Zhang , V. Geoffroy , A. L. Ridall , and G. Karsenty , “Osf2/Cbfa1: A Transcriptional Activator of Osteoblast Differentiation,” Cell 89 (1997): 747–754, 10.1016/s0092-8674(00)80257-3. 9182762

[30] S. Zhu , W. Chen , A. Masson , and Y. P. Li , “Cell Signaling And Transcriptional Regulation Of Osteoblast Lineage Commitment, Differentiation, Bone Formation, And Homeostasis,” Cell Discovery 10 (2024): 71, 10.1038/s41421-024-00689-6. 38956429 PMC11219878

[31] K. L. Lin , C. H. Chou , S. C. Hsieh , S. Y. Hwa , M. T. Lee , and F. F. Wang , “Transcriptional Upregulation of DDR2 by ATF4 Facilitates Osteoblastic Differentiation Through p38 MAPK‐Mediated Runx2 Activation,” Journal of Bone and Mineral Research 25 (2010): 2489–2503, 10.1002/jbmr.159. 20564243

[32] S. A. Chae , J. S. Son , M. J. Zhu , J. M. De Avila , and A. M. Du , “Treadmill Running of Mouse as a Model for Studying Influence of Maternal Exercise on Offspring,” Bio‐Protocol 10 (2020): 3838, 10.21769/BioProtoc.3838. PMC7842301 33659487

[33] J. S. Son , L. Zhao , Y. Chen , et al., “Maternal Exercise Via Exerkine Apelin Enhances Brown Adipogenesis And Prevents Metabolic Dysfunction In Offspring Mice,” Science Advances 6 (2020): aaz0359, 10.1126/sciadv.aaz0359. PMC7164955 32494609

[34] X. Zhou , R. Li , J. J. Michal , et al., “Accurate Profiling of Gene Expression and Alternative Polyadenylation With Whole Transcriptome Termini Site Sequencing (WTTS‐Seq),” Genetics 203 (2016): 683–697, 10.1534/genetics.116.188508. 27098915 PMC4896187

[35] Y. T. Chen , Y. Hu , Q. Y. Yang , et al., “Excessive Glucocorticoids During Pregnancy Impair Fetal Brown Fat Development and Predispose Offspring to Metabolic Dysfunctions,” Diabetes 69 (2020): 1662–1674, 10.2337/db20-0009. 32409491 PMC7372078

[36] X. S. Pan , J. Li , E. B. Brown , and C. K. Kuo , “Mechanics of development ‐ PMC ‐ NIH,” Philosophical Transactions of the Royal Society of London Series B, Biological Sciences 373 (2018): 1759, 10.1098/rstb.2017.0325.

[37] N. C. Nowlan , P. Murphy , and P. J. Prendergast , “Mechanobiology of Embryonic Limb Development,” Annals of the New York Academy of Sciences 1101 (2007): 389–411, 10.1196/annals.1389.003. 17344536

[38] N. C. Nowlan , J. Sharpe , K. A. Roddy , P. J. Prendergast , and P. Murphy , “Mechanobiology of Embryonic Skeletal Development: Insights From Animal Models,” Birth Defects Research Part C: Embryo Today: Reviews 90 (2010): 203–213, 10.1002/bdrc.20184. 20860060 PMC4794623

[39] V. Arvind and A. H. Huang , “Mechanobiology of Limb Musculoskeletal Development,” Annals of the New York Academy of Sciences 1409 (2017): 18–32, 10.1111/nyas.13427. 28833194 PMC5730481

[40] Y. Kim , H. A. Parry , T. B. Willingham , et al., “Reorganization of Mitochondria–Organelle Interactions During postnatal Development in Skeletal Muscle,” The Journal of Physiology 602 (2024): 891–912, 10.1113/jp285014. 38429930 PMC10939894

[41] H. Kaji , “Interaction Between Muscle and Bone,” Journal of Bone Metabolism 21 (2014): 29, 10.11005/jbm.2014.21.1.29. 24707465 PMC3970293

[42] S. Rahman and W. C. Copeland , “POLG‐Related Disorders And Their Neurological Manifestations,” Nature Reviews Neurology 15 (2019): 40–52, 10.1038/s41582-018-0101-0. 30451971 PMC8796686

[43] A. H. Schapira , “Mitochondrial Disease,” The Lancet 368 (2006): 70–82, 10.1016/s0140-6736(06)68970-8. 16815381

[44] T. H. Chen , K. Y. Koh , K. M. Lin , and C. K. Chou , “Mitochondrial Dysfunction as an Underlying Cause of Skeletal Muscle Disorders,” International Journal of Molecular Sciences 23 (2022): 12926, 10.3390/ijms232112926. 36361713 PMC9653750

[45] K. G. Avin and R. N. Moorthi , “Bone is Not Alone: The Effects of Skeletal Muscle Dysfunction in Chronic Kidney Disease,” Current Osteoporosis Reports 13 (2015): 173–179, 10.1007/s11914-015-0261-4. 25691218 PMC4860015

[46] A. G. Berman , J. M. Organ , M. R. Allen , and J. M. Wallace , “Muscle Contraction Induces Osteogenic Levels Of Cortical Bone Strain Despite Muscle Weakness In A Mouse Model of Osteogenesis Imperfecta,” Bone 132 (2020): 115061, 10.1016/j.bone.2019.115061. 31805389 PMC7720097

[47] L. Han , Y. Chen , L. Li , et al., “Increased mtDNA Mutation Frequency In Oocytes Causes Epigenetic Alterations And Embryonic Defects,” National Science Review 9 (2022): nwac136, 10.1093/nsr/nwac136. 36325113 PMC9616472

[48] Y. Chen , G. Ma , Y. Gai , et al., “AMPK Suppression Due to Obesity Drives Oocyte mtDNA Heteroplasmy via ATF5‐POLG Axis,” Advanced Science 11 (2024): 2307480, 10.1002/advs.202307480. 38499990 PMC11132083

[49] J. S. Son , S. A. Chae , Y. H. Chun , H. Wang , Z. Jiang , and M. Du , “High‐Calorie Diet During Pregnancy Leads to Muscular Fibrosis and Neuromuscular Damage in Offspring Mice,” Journal of Cachexia, Sarcopenia And Muscle 16 (2025): 70027, 10.1002/jcsm.70027. PMC12440564 40957424

[50] H. Suominen , “Muscle Training For Bone Strength,” Aging Clinical and Experimental Research 18 (2006): 85, 10.1007/bf03327422. 16702776

[51] L. J. Moyer‐Mileur , S. D. Ball , V. L. Brunstetter , and G. M. Chan , “Maternal‐Administered Physical Activity Enhances Bone Mineral Acquisition In Premature Very Low Birth Weight Infants,” Journal of Perinatology 28 (2008): 432–437, 10.1038/jp.2008.17. 18337741

[52] A. Safdar , J. M. Bourgeois , D. I. Ogborn , et al., “RETRACTED: Endurance Exercise Rescues Progeroid Aging And Induces Systemic Mitochondrial Rejuvenation in mtDNA Mutator Mice,” Proceedings of the National Academy of Sciences 108 (2011): 4135–4140, 10.1073/pnas.1019581108. PMC3053975 21368114

[53] J. Clark‐Matott , A. Saleem , Y. Dai , et al., “Metabolomic Analysis Of Exercise Effects in the POLG Mitochondrial DNA Mutator Mouse Brain,” Neurobiology of Aging 36 (2015): 2972–2983, 10.1016/j.neurobiolaging.2015.07.020. 26294258 PMC4609600

[54] C. Faraci , S. Annis , J. Jin , H. Li , K. Khrapko , and D. C. Woods , “Impact of Exercise On Oocyte Quality in the POLG Mitochondrial DNA Mutator Mouse,” Reproduction 156 (2018): 185–194, 10.1530/rep-18-0061. 29875308 PMC6074767

[55] B. C. Frier , D. B. Williams , and D. C. Wright , “R1761,” American Journal of Physiology Regulatory, Integrative and Comparative Physiology 297 (2009): R1761–R1768, 10.1152/ajpregu.00422.2009. 19793954

[56] A. Than , H. L. He , S. H. Chua , et al., “Apelin Enhances Brown Adipogenesis and Browning of White Adipocytes,” Journal of Biological Chemistry 290 (2015): 14679–14691, 10.1074/jbc.M115.643817. 25931124 PMC4505534

[57] J. S. Son , X. Liu , Q. Tian , et al., “Exercise Prevents The Adverse Effects Of Maternal Obesity On Placental Vascularization And Fetal Growth,” The Journal of Physiology 597 (2019): 3333–3347, 10.1113/jp277698. 31115053 PMC6602814

[58] S. A. Chae , J. S. Son , J. M. de Avila , M. Du , and M. J. Zhu , “Maternal Exercise Improves Epithelial Development Of Fetal Intestine By Enhancing Apelin Signaling And Oxidative Metabolism,” American Journal of Physiology‐Regulatory, Integrative and Comparative Physiology 323 (2022): R728–R738, 10.1152/ajpregu.00128.2022. 36189989 PMC9829469

[59] M. J. Miller , G. R. Marcotte , N. Basisty , et al., “The Transcription Regulator ATF4 is a Mediator Of Skeletal Muscle Aging,” GeroScience 45 (2023): 2525–2543, 10.1007/s11357-023-00772-y. 37014538 PMC10071239

[60] H. T. Le , J. Yu , H. S. Ahn , et al., “eIF2α phosphorylation‐ATF4 Axis‐Mediated Transcriptional Reprogramming Mitigates Mitochondrial Impairment During ER stress,” Molecules and Cells 48 (2025): 100176, 10.1016/j.mocell.2024.100176. 39756584 PMC11786836

