# Supplementary material for: Maternal Exercise Rescues Embryonic Osteogenesis Impaired due to POLG Mutation Through a Potential Apelin‐ATF4 Axis
Source: Adv Sci (Weinh). 2026 Aug 7;13(60):e77029. doi: 10.1002/advs.77029 (PMC13449544; doi:10.1002/advs.77029)
Supplement: Supplementary file 1 [file ADVS-13-e77029-s001.pdf]

Supplementary materials

Maternal exercise rescues embryonic osteogenesis impaired due to POLG mutation through a potential apelin-ATF4 axis

Song Ah Chae, Ryan C. Riddle, Zhihua Jiang, Chaeyoung Shin, Min Du, Jun Seok Son

Supplementary Table 1. Primer sequences used for qPCR analysis.

Gene Name	Forward/Reverse	Primer sequence
Ocn	F	5'-ACAGACTCCGGCGCTACCTT-3'
	R	5'-AATAGTGATACCGTAGATGCGTTTG-3'
Runx2	F	5'-CATTTGCACTGGGTCACACGTA-3'
	R	5'-GAATCTGGCCATGTTTGTGCTC-3'
Osx	F	5'-ACTCATCCCTATGGCTCGTG-3'
	R	5'-GGTAGGGAGCTGGGTTAAGG-3'
Rankl	F	5'-TGCAGAAGGAACTGCAACAC-3'
	R	5'-GATGGTGAGGTGTGCAAATG-3'
Opg	F	5'-ACAGCTGGCACACCAGTGAT-3'
	R	5'-ATCCTCTCTACACTCTCGGC-3'
Ctsk	F	5'-CAGCAGAACGGAGGCATTGA-3'
	R	5'-CTTTGCCGTGGCGTTATACATAC-3'
Trap	F	5'-CAGCAGCCAAGGAGGACTAC-3'
	R	5'-ACATAGCCCACACCGTTCTC-3'
Myf5	F	5'-AAACTCCGGGAGCTCCGCCT-3'
	R	5-GGCAGCCGTCCGTCATGTCC-3'
Pax7	F	5'-TTGGGGAACACTCCGCTGTGC-3'
	R	5'-CAGGGCTTGGGAAGGGTTGGC-3'
Myod1	F	5'-TCTGGAGCCCTCCTGGCACC-3'
	R	5'-CGGGAAGGGGGAGAGTGGGG-3'
Myog	F	5'-GAGATCCTGCGCAGCGCCAT-3'
	R	5'-CCCCGCCTCTGTAGCGGAGA-3'
Pgc1a	F	5'-AGCCGTGACCACTGACAACGAG-3'
	R	5'-GCTGCATGGTTCTGAGTGCTAAG-3'
Pparg	F	5'-GCATGGTGCCTTCGCTGA-3'
	R	5'-TGGCATCTCTGTGTCAACCATG-3'
Tfam	F	5'-CCAAAAAGACCTCGTTCAGC-3'
	R	5'-CTTCAGCCATCTGCTCTTCC-3'
Nrf1	F	5'-GCACCTTTGGAGAATGTGGT-3'
	R	5'-CTGAGCCTGGGTCATTTTGT-3'
Apln	F	5'-GTTTGTGGAGTGCCACTG-3'
	R	5'-CGAAGTTCTGGGCTTCAC-3'
Apj	F	5'-GCTGTGCCTGTCATGGTGTT-3'
	R	5'-CACTGGATCTTGGTGCCATTT-3'
Actb	F	5'-TGTCCACCTTCCAGCAGATGT-3'
	R	5'-AGCTCAGTAACAGTCCGCCTAG-3'
18S	F	5'-GTAACCCGTTGAACCCCATT-3'
	R	5'-CCATCCAATCGGTAGTAGCG-3'
Pgc1a-MeDIP	F	5'-TTCCTCTCTCTCTAAGCGTTACTTCA-3'
	R	5'-CTTACTACAGTCCCCAGTCACATGA-3'

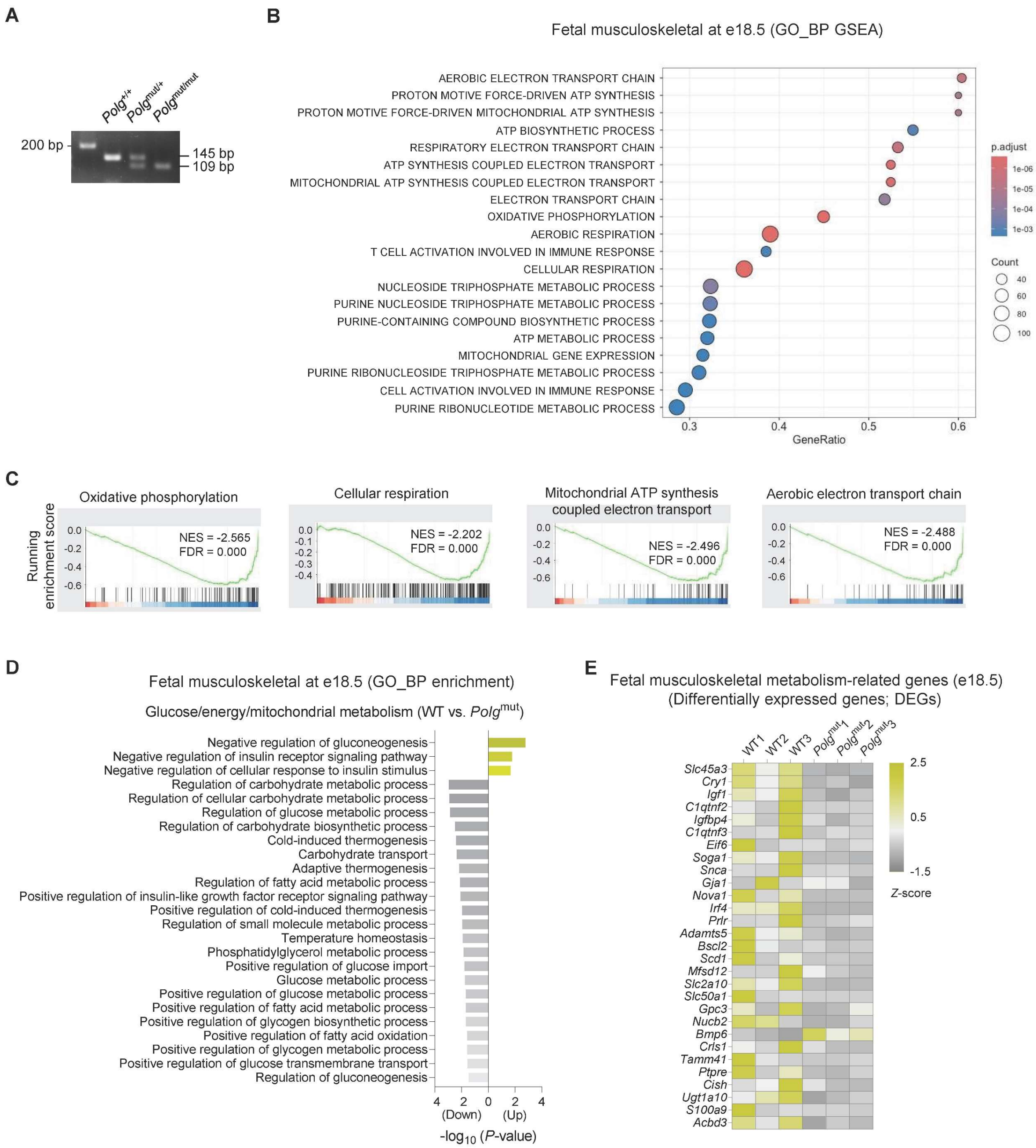

Supplementary Figure 1. POLG mutation alters fetal musculoskeletal metabolic and mitochondrial transcriptional regulations at e18.5. A, Representative genotyping PCR image used to identify fetal genotypes. **B,** Gene ontology (GO) biological process GSEA of fetal musculoskeletal tissues at e18.5 comparing WT and *Polg*^{mut} groups. Dot size represents gene count, and color indicates adjusted *P* value. **C,** Representative enrichment plots for selected GO biological process pathways, including oxidative

phosphorylation, cellular respiration, mitochondrial ATP synthesis coupled electron transport, and aerobic electron transport chain. Normalized enrichment score (NES) and false discovery rate (FDR) are indicated in each plot. **D,** GO biological process enrichment analysis of DEGs related to glucose, energy, and mitochondrial metabolism in fetal musculoskeletal tissues at e18.5 comparing WT and $Polg^{mut}$ groups. **E,** Heatmap of selected metabolism related differentially expressed genes in fetal musculoskeletal tissues at e18.5. Each column represents an individual sample and each row represents a gene. Relative expression levels are shown as *Z*-scores.

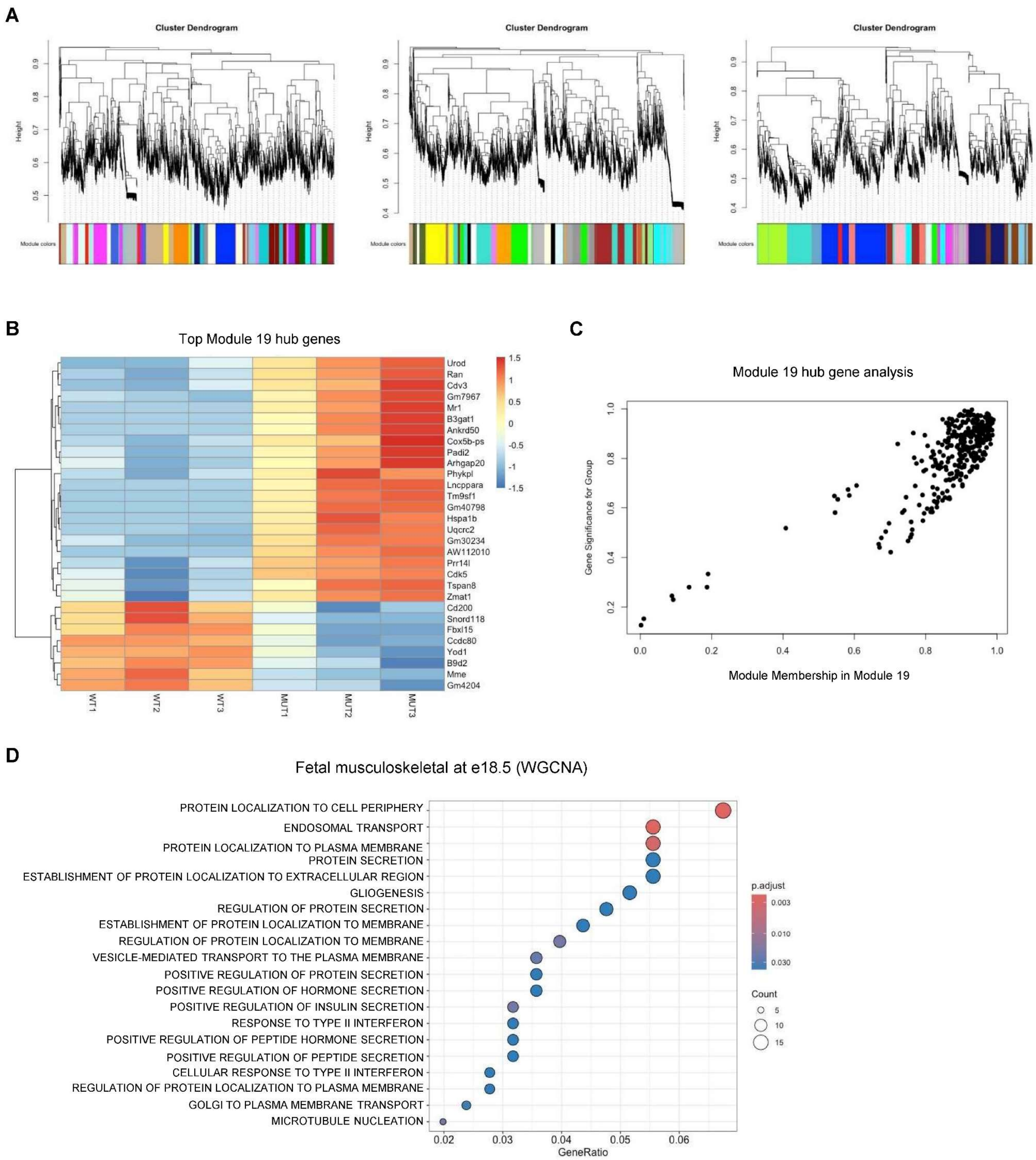

Supplementary Figure 2. WGCNA identifies the ME19 module and its associated biological pathways in fetal musculoskeletal tissues at e18.5. A, Cluster dendrograms showing gene modules identified by weighted gene co-expression network analysis (WGCNA) in fetal musculoskeletal tissues at e18.5. Module colors are indicated below each dendrogram. **B,** Heatmap of the top hub genes in the Module 19 module across WT and *Polg*^{mut} samples. Relative expression levels are shown as *Z*-scores. **C,** Scatter plot of hub

gene analysis for the Module 19 module, showing the relationship between module membership and gene significance for the genotype group. **D,** Functional enrichment analysis of genes within the Module 19 module from fetal musculoskeletal tissues at e18.5. Dot size represents gene count, and color indicates adjusted *P* value.

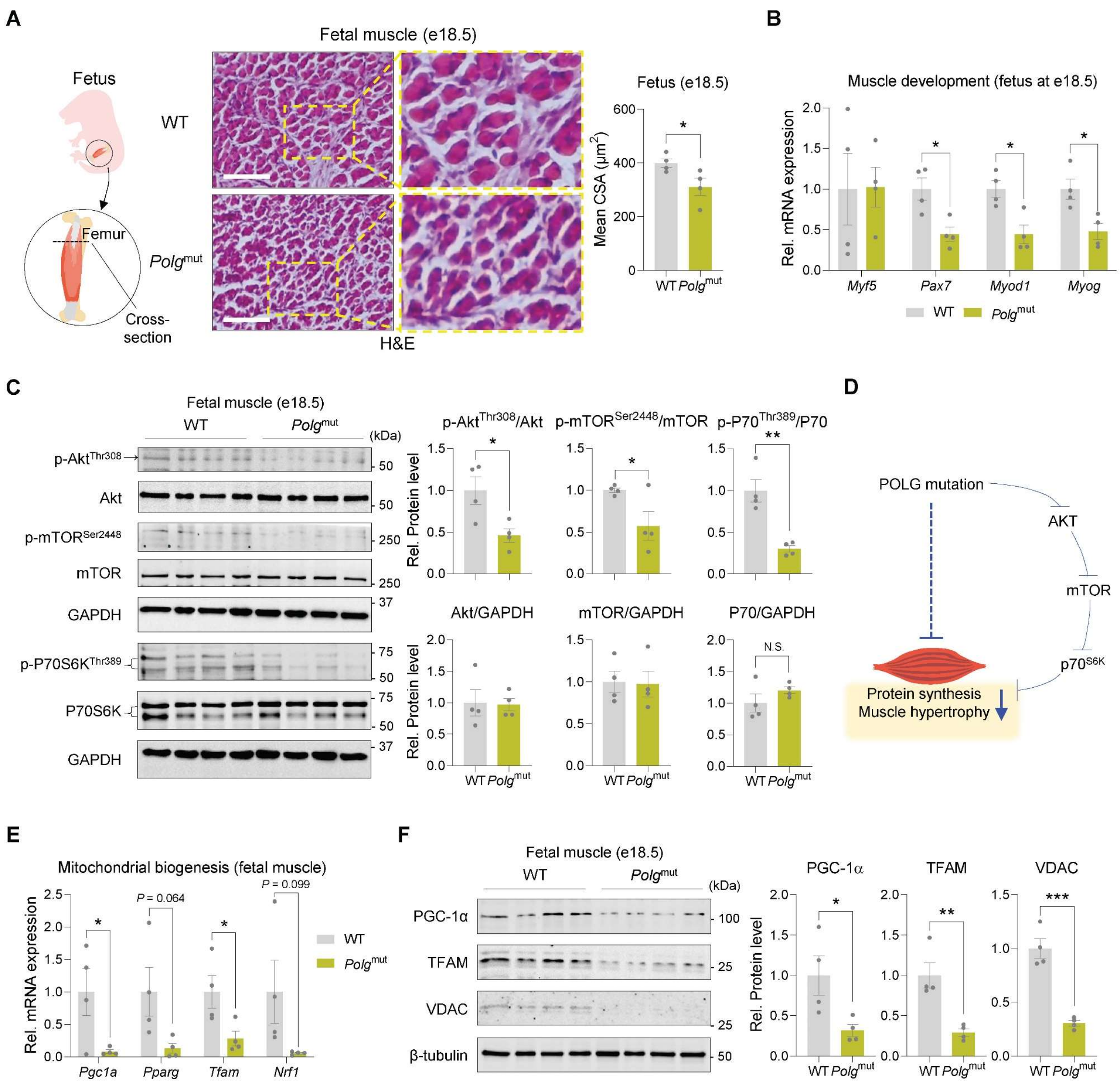

Supplementary Figure 3. POLG mutation impedes fetal muscle development. A, Representative H&E images and means of cross-sectional areas (CSAs) in fetal muscle (Scale bar = 100 μm). **B,** Relative mRNA expression of myogenic genes (n = 4). **C, D,** Cropped western blot images and relative protein levels (C, n = 4) and diagram (D) of POLG mutation-dependent muscle hypertrophy signaling. **E,** Relative mRNA expression of mitochondrial biogenic genes in POLG mutated fetal muscle (n = 4). **F,** Cropped western blot analysis of mitochondrial biogenesis in POLG mutated fetal muscle (n = 4/group). Target proteins were normalized by their total proteins or GAPDH. Mean ± s.e.m., and each dot represents one litter. *$P < 0.05$, **$P < 0.01$, and ***$P < 0.001$ in WT vs. *Polg*^{mut} by two-sided *P* values by Student *t*-test (B-F).

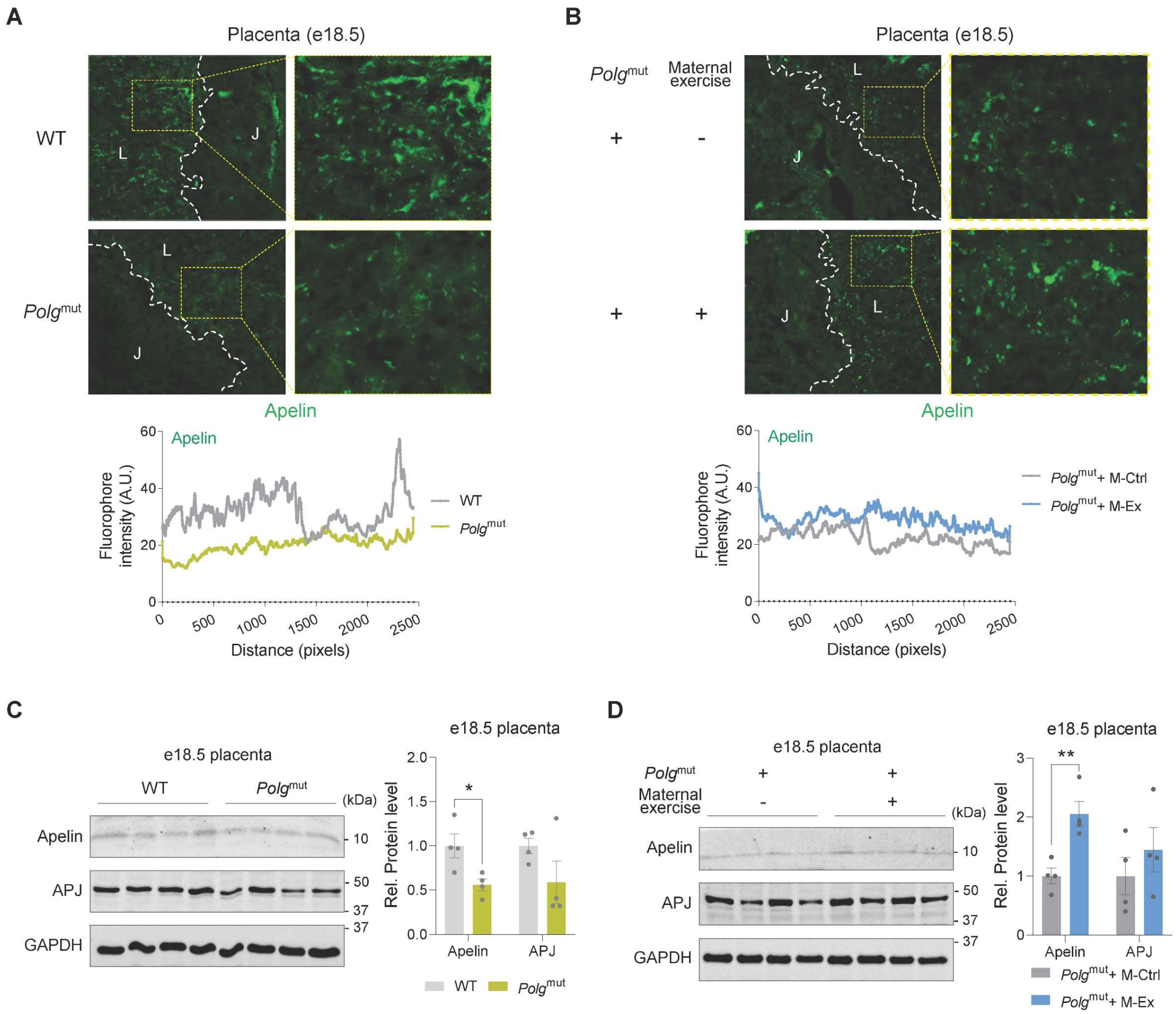

Supplementary Figure 4. Maternal exercise restores placental apelin signaling in POLG mutation at e18.5. A, B, Representative IF images of apelin in placental sections at e18.5 from WT and *Polg*^{mut} (A) or *Polg*^{mut} +M-Ctrl and *Polg*^{mut} +M-Ex (B) groups, with corresponding fluorescence intensity profiles across the indicated region (L, labyrinth; J, junctional zone). **C, D,** Cropped western blot images and relative protein levels of apelin and APJ from WT and *Polg*^{mut} (C) or *Polg*^{mut} +M-Ctrl and *Polg*^{mut} +M-Ex (D) groups (n = 4/group). Target proteins were normalized by GAPDH. Mean ± s.e.m., and each dot represents one litter. **P* < 0.05 and ***P* < 0.01 in WT vs. *Polg*^{mut} or *Polg*^{mut}+M-Ctrl vs. *Polg*^{mut}+M-Ex by two-sided *P* values by Student *t*-test (C, D).

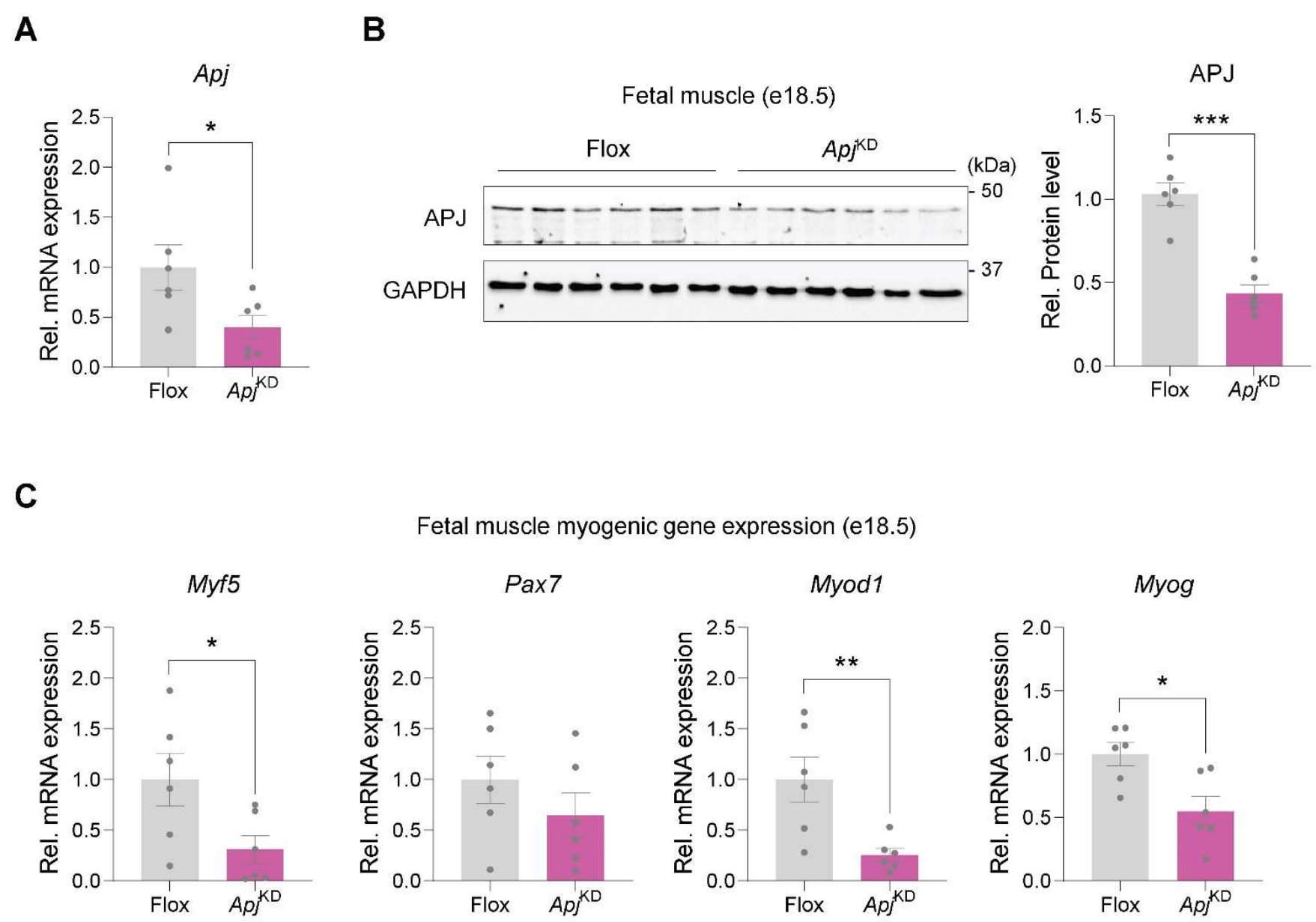

Supplementary Figure 5. APJ knockdown reduces APJ expression and myogenic signaling in fetal muscle at e18.5. A, Relative mRNA expression of *Apj* in fetal muscle. **B,** Cropped immunoblots and quantification of APJ protein levels in fetal muscle from Flox and Apj^{KD} fetuses. **C,** Relative mRNA expression of fetal myogenic genes, including *Myf5*, *Pax7*, *Myod1*, and *Myog*, in fetal muscle. Data are presented as mean ± s.e.m. Each dot represents one litter. *$P < 0.05$, **$P < 0.01$, and ***$P < 0.001$ in Flox vs. Apj^{KD} by two-sided *P* values by Student *t*-test (A-C).

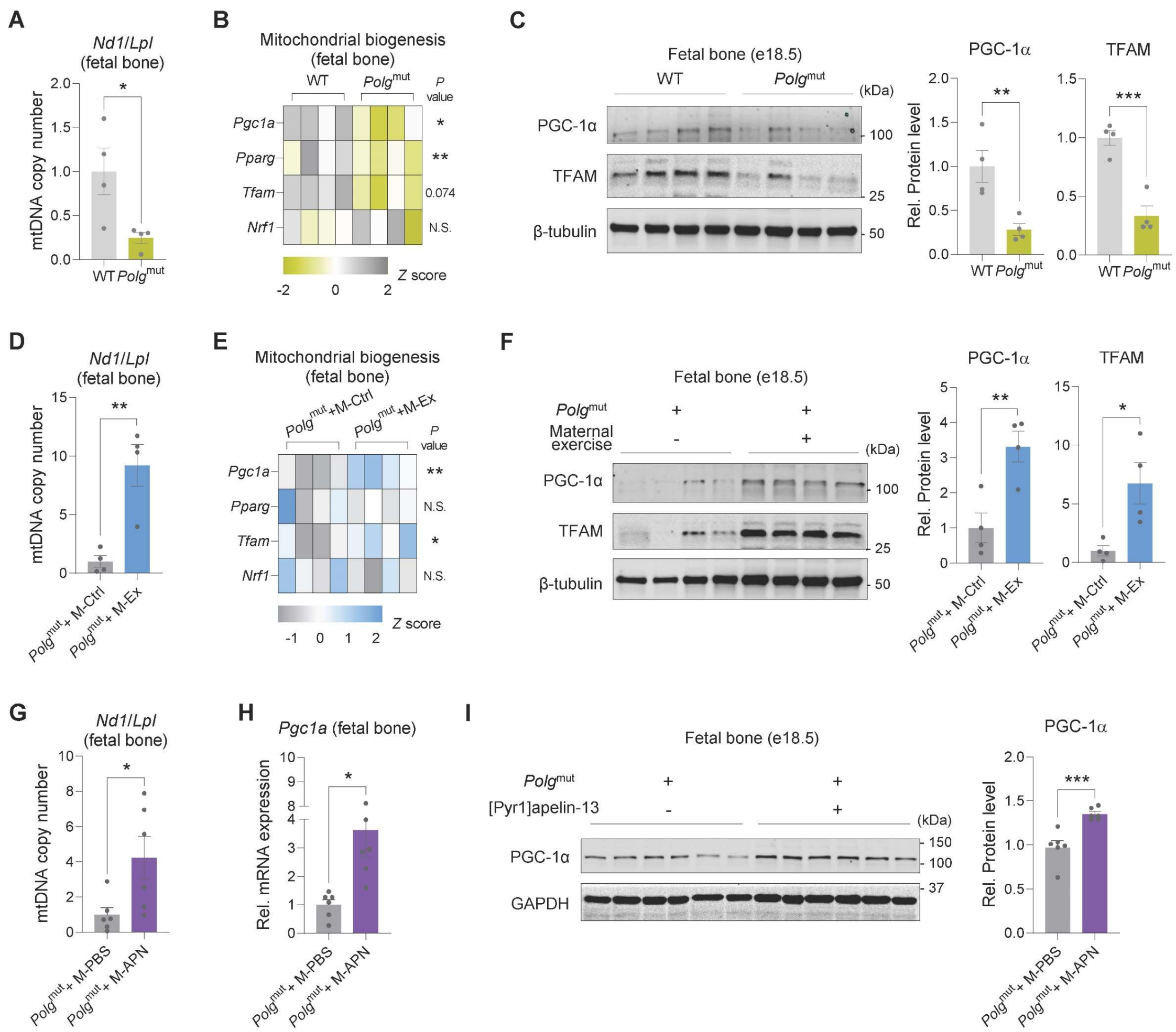

Supplementary Figure 6. ME-apelin signaling pathway improve mitochondrial biogenesis in POLG mutated fetal bone. A, D, G, Mitochondrial DNA copy number in fetal bone from WT and *Polg*^{mut} (A; n = 4), *Polg*^{mut} +M-Ctrl and *Polg*^{mut} +M-Ex (D; n = 4), or *Polg*^{mut} +M-PBS and *Polg*^{mut} +M-APN (G; n = 6) groups. **B, E, H,** Relative mRNA expression of mitochondrial biogenic genes in fetal from WT and *Polg*^{mut} (B; n = 4), *Polg*^{mut} +M-Ctrl and *Polg*^{mut} +M-Ex (E; n = 4), or *Polg*^{mut} +M-PBS and *Polg*^{mut} +M-APN (H; n = 6) groups. **C, F, I,** Cropped western blot analyses of mitochondrial biogenic genes in fetal bone from WT and *Polg*^{mut} (C; n = 4), *Polg*^{mut} +M-Ctrl and *Polg*^{mut} +M-Ex (F; n = 4), or *Polg*^{mut} +M-PBS and *Polg*^{mut} +M-APN (I; n = 6) groups. Target proteins were normalized by β-tubulin or GAPDH. Mean ± s.e.m., and each dot represents one litter. *$P < 0.05$, **$P < 0.01$, and ***$P < 0.001$ in WT vs. *Polg*^{mut}, *Polg*^{mut}+M-Ctrl vs. *Polg*^{mut}+M-Ex, or *Polg*^{mut}+M-PBS vs. *Polg*^{mut}+M-APN by two-sided *P* values by Student *t*-test (A-I).

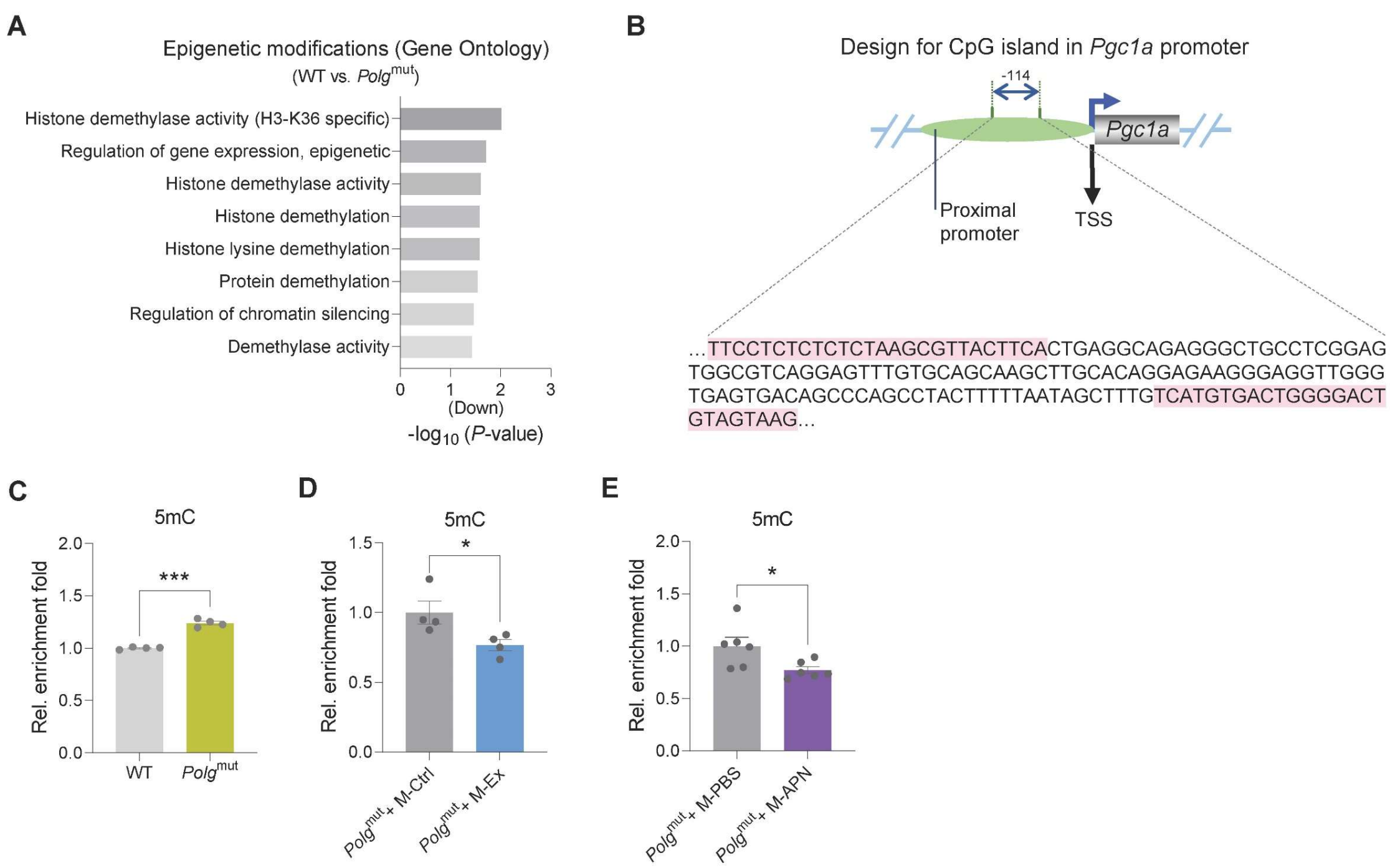

Supplementary Figure 7. ME-apelin-mediated epigenetic regulations of *Pgc1a* promoter in POLG mutant fetal bone at e18.5. A, GO analysis in POLG mutated fetal musculoskeletal, generated from RNA-seq analysis. **B,** Design of CpG island of *Pgc1a* promoter. **C-E,** Relative enrichment folds of 5-methylcytosine (5mC) in POLG mutation (C, n = 4), POLG mutation following ME (D, n = 4), and POLG mutation with maternal apelin administration (E, n = 6). Mean ± s.e.m., and each dot represents one litter. *$P < 0.05$ and ***$P < 0.001$ in WT vs. *Polg*^{mut}, *Polg*^{mut}+M-Ctrl vs. *Polg*^{mut}+M-Ex, or *Polg*^{mut}+M-PBS vs. *Polg*^{mut}+M-APN by two-sided *P* values by Student *t*-test (C-E).
